# c-Myc and AMPK Control Cellular Energy Levels by Cooperatively Regulating Mitochondrial Structure and Function

**DOI:** 10.1371/journal.pone.0134049

**Published:** 2015-07-31

**Authors:** Lia R. Edmunds, Lokendra Sharma, Huabo Wang, Audry Kang, Sonia d’Souza, Jie Lu, Michael McLaughlin, James M. Dolezal, Xiaoli Gao, Susan T. Weintraub, Ying Ding, Xuemei Zeng, Nathan Yates, Edward V. Prochownik

**Affiliations:** 1 Section of Hematology/Oncology, Children’s Hospital of Pittsburgh of UPMC, Pittsburgh, PA, United States of America; 2 The University of Pittsburgh School of Medicine, Pittsburgh, PA, United States of America; 3 Department of Biochemistry, The University of Texas Health Science Center at San Antonio, San Antonio TX, United States of America; 4 Department of Biostatistics, The University of Pittsburgh, Pittsburgh, PA, United States of America; 5 Department of Cell Biology, The University of Pittsburgh School of Medicine, Pittsburgh, PA, United States of America; 6 Department of Microbiology and Molecular Genetics, The University of Pittsburgh School of Medicine, Pittsburgh, PA, United States of America; 7 The Hillman Cancer Center, The University of Pittsburgh, Pittsburgh, PA, United States of America; University of South Alabama Mitchell Cancer Institute, UNITED STATES

## Abstract

The c-Myc (Myc) oncoprotein and AMP-activated protein kinase (AMPK) regulate glycolysis and oxidative phosphorylation (Oxphos) although often for different purposes. Because Myc over-expression depletes ATP with the resultant activation of AMPK, we explored the potential co-dependency of and cross-talk between these proteins by comparing the consequences of acute Myc induction in *ampk+/+* (WT) and *ampk-/-* (KO) murine embryo fibroblasts (MEFs). KO MEFs showed a higher basal rate of glycolysis than WT MEFs and an appropriate increase in response to activation of a Myc-estrogen receptor (MycER) fusion protein. However, KO MEFs had a diminished ability to increase Oxphos, mitochondrial mass and reactive oxygen species in response to MycER activation. Other differences between WT and KO MEFs, either in the basal state or following MycER induction, included abnormalities in electron transport chain function, levels of TCA cycle-related oxidoreductases and cytoplasmic and mitochondrial redox states. Transcriptional profiling of pathways pertinent to glycolysis, Oxphos and mitochondrial structure and function also uncovered significant differences between WT and KO MEFs and their response to MycER activation. Finally, an unbiased mass-spectrometry (MS)-based survey capable of quantifying ~40% of all mitochondrial proteins, showed about 15% of them to be AMPK- and/or Myc-dependent in their steady state. Significant differences in the activities of the rate-limiting enzymes pyruvate kinase and pyruvate dehydrogenase, which dictate pyruvate and acetyl coenzyme A abundance, were also differentially responsive to Myc and AMPK and could account for some of the differences in basal metabolite levels that were also detected by MS. Thus, Myc and AMPK are highly co-dependent and appear to engage in significant cross-talk across numerous pathways which support metabolic and ATP-generating functions.

## Introduction

c-Myc (Myc) oncoprotein de-regulation occurs in a substantial fraction of human cancers and alters numerous transformation-associated phenotypes [1–4]. Myc over-expression exerts marked effects on proliferation, survival, differentiation and biomass accumulation as a result of global changes in the expression of RNAs regulated by all 3 RNA polymerases [1, 5–8]. Together, these changes reflect Myc’s role as a general transcription factor that broadly modulates the levels of most, if not all, genes [9–12]. The molecular mechanisms by which Myc mediates these effects on transcription are varied and highly dependent upon the degree of Myc over-expression, the identity of various co-factors and the type of cell in which Myc de-regulation occurs [6, 11, 13, 14].

Metabolic changes are among the most universal consequences of aberrant Myc expression [15]. Myc induces the majority of genes encoding glycolytic enzymes and thus is important for promoting the Warburg effect, defined as the persistence of glycolysis under aerobic conditions [5, 16]. Rather than being a result of defective mitochondrial function and confined to tumor cells as originally proposed [17], the Warburg effect also occurs in rapidly proliferating normal cells [16, 18]. It thus seems likely that the major purpose of the Warburg effect is to supply anabolic precursors such as ribose sugars, nucleotides and select amino acids whose production must be increased and carefully coordinated with the doubling of biomass that accompanies replication [16, 19].

In addition to enhancing glycolysis, Myc re-programs oxidative phosphorylation (Oxphos) and supports the structural and functional integrity of mitochondria and the electron transport chain (ETC) via the direct up-regulation of certain mitochondrial-specific transcription factors [20, 21]. This has the effect of increasing the production of ATP needed to support macromolecular synthesis during proliferation [15, 20, 22]. Concurrently, Myc promotes the uptake and β-oxidation of exogenous fatty acids, which serve as an alternate source of acetyl CoA that is otherwise provided in lower yield by the re-programmed glycolytic pathway [23–25]. The transport of glutamine and its conversion to glutamate and α-ketoglutarate are also under stringent positive Myc control and provide yet another source of TCA cycle intermediates [5, 15, 26]. In support of all the above findings, *myc-/-* fibroblasts show severe structural and functional ETC defects, low rates of glycolysis and Oxphos and profound ATP depletion [20].

The regulation of both anabolic and catabolic processes is also a property of AMP-activated protein kinase (AMPK), a Ser/Thr kinase that is activated in response to a decrease in the ATP: AMP ratio [27–29]. AMPK is a trimeric enzyme whose γ-regulatory subunit undergoes a conformational change upon binding AMP that allows phosphorylation of the α catalytic subunit’s Thr_172_ residue by the upstream kinase and putative tumor suppressor LKB [27, 30, 31]. The consequences of this activating phosphorylation event include a general inhibition of energy-consuming processes such as protein and fatty acid synthesis and proliferation [27] and an increase in energy-generating process such as glycolysis and Oxphos [27, 32, 33]. Collectively, these cooperate to ensure the timely restoration of a positive ATP: AMP balance and allow the resumption of proliferation. Thus AMPK and Myc appear to enhance energy-generating processes while simultaneously exerting opposing effects on energy-consuming processes. How these are regulated and coordinated remain largely unexplored.

We have recently observed that the ATP-depleted state of *myc-/-* fibroblasts is associated with chronic phosphorylation-dependent AMPK activation whereas Myc re-expression restores normal ATP levels and suppresses AMPK [23]. These observations suggest that Myc and AMPK engage in a form of cross-talk, the purpose of which is to optimize proliferation and energy production while balancing Oxphos and the Warburg effect. In the current work, we have investigated how and the extent to which such communication occurs as well as how compromising AMPK function affects Myc’s metabolic phenotype.

## Materials and Methods

### Cell culture

SV40 T-antigen-immortalized *ampk+/+* and *ampk-/-* MEFs, the latter bearing a double knockout of the α1 and α2 subunits of AMPK, were a kind gift from Dr. Benoit Viollet (Institut Cochin, Université Paris Descartes) and Dr. Keith Laderoute (Discovery Technologies, SRI International) [34, 35] and were generated as described by Laderoute *et al*. [36]. Both cell lines were transduced with a pBabePuro retroviral vector encoding a Myc-estrogen receptor (MycER) fusion protein [37]. Stable clones of each AMPK genotype (hereafter referred to as WT or KO) were selected in 1 μg/ml of puromycin, pooled, and used for all subsequent experiments. Both WT and KO cells expressed equivalent levels of MycER (S1A Fig). All cell lines were maintained in puromycin-containing Dulbecco’s-modified Eagle’s minimal essential medium (D-MEM) supplemented with 10% heat-inactivated fetal bovine serum (FBS), L-glutamine and penicillin/streptomycin as previously described [20]. Unless otherwise stated, MycER was activated by adding 4-hydroxytamoxifen (4HT) to cells for 7–9 days at a final concentration of 250 nM before performing any assessments. All recombinant DNA and retroviral and lentiviral work was approved by the University of Pittsburgh Recombinant DNA and Institutional Biosafety Committees and, in the latter cases, was performed under BSL2+ conditions.

### Quantification of glycolysis, Oxphos and ATP levels

All experiments were performed on an XF24 Extracellular Flux Analyzer (Seahorse Bioscience, Billirica, MA) as previously described [20, 23]. O_2_ consumption rate (OCR) and proton production, expressed as the extracellular acidification rate (ECAR), were quantified in unbuffered D-MEM containing 8.3 g of glucose- and pyruvate-free DMEM (Sigma) supplemented with 31 mM NaCl, 2 mM glutamine, 42.3 μM phenol red, and 25 mM glucose, pH 7.4 to obtain baseline metabolic levels. A mitochondrial stress-test was applied by adding 1 μM oligomycin, 0.3 μM FCCP, 100 mM 2-deoxyglucose (2-DG), and 1 μM rotenone. Each measurement point was performed in quadruplicate and experiments were repeated at least 3 times with similar results and normalized to cell number at the conclusion of the experiment. Relative effects were expressed as areas under the curve measurements that were generated by the manufacturer’s software.

ATP assays were performed on 20,000–30,000 cells seeded in 96 well plates the day before and were performed in quadruplicate wells using the ATPlite Luminescence Assay System (Perkin Elmer, Waltham, MA) as instructed by the manufacturer. Results were normalized to total protein levels, which were determined on separate sets of identical wells.

### Measurements of mitochondrial mass and reactive oxygen species (ROS)

Mitochondrial mass was determined as previously described [20, 23]. Monolayers were stained at 37C for 45 min in fresh D-MEM containing 20 nM of acridine orange 10-nonyl bromide (NAO), 0.5 mM of MitoTracker Green, 10 μM of CM-H2-DCFDA or 5 μM of MitoSox (all from Invitrogen, Carlsbad, CA)) and then analyzed using a FACStar flow cytometer (Becton-Dickinson Biosciences, San Jose, CA). Analyses were performed using BD FACSDiva Software as previously described [20].

### Blue native gel electrophoresis (BNGE) and electron transport chain (ETC) enzyme assays

Samples were prepared for BNGE as previously described with some modifications [20]. Cells were suspended in 0.5 ml of ice cold HB buffer (50 mM KPO_4_, pH = 7.4; 1mM EDTA; 2.5% glycerol; 250 mM sucrose) containing protease inhibitor cocktail (Sigma-Aldrich, St. Louis, MO), disrupted on ice with a dounce homogenizer (Isobiotec, Heidelberg, Germany) and enriched for mitochondria by differential centrifugation. The pellet was washed twice with HB buffer and re-suspended in the same buffer at a final protein concentration of 2–5 mg/ml. To achieve optimal solubility of mitochondrial super-complexes/complexes, the digitonin concentration was optimized so that 8 mg of digitonin was added per mg of protein in HB buffer without EDTA. Following a 20 min incubation on ice, a Coomassie blue solution (5% Coomassie blue G250 in 750 mM 6-aminocaproic acid) was added (1:30 v/v). The supernatant was then electrophoresed on a 3–12% Native PAGE Novex Bis-Tris gel (Invitrogen) at 80 V for 4 hours at 4C in the buffer provided by the supplier. 80 μg of protein for each sample was electrophoresed to resolve complexes. Following electrophoresis, gels were stained for 30 min with Bio-Safe Coomassie G250 (Bio-Rad, Hercules, CA). Gels were scanned and the images analyzed for relative band density using AlphaEaseFC 2200 scanner and AlphaEaseFC software.

*In situ* gel assays of individual ETC complex activities were performed as previously described for Complexes I (NADH ubiquinone oxidoreductase) and V (ATPase) [38]. Complex III (CIII) (decylubiquinol cytochrome c oxidoreductase) was assayed by incubating gels with CIII assay solution [39] overnight with mild agitation. In a separate reaction, Complex IV (CIV) (cytochrome c oxidase) was measured by incubating the gel in a solution containing 1 nM catalase, 10 mg cytochrome c and 750 mg sucrose in CIII assay buffer with mild agitation for 30 min. An optional wash in water at room temperature was employed for 24 hours to further sharpen the band patterns. Band intensities were quantified using NIH Image J software and were normalized with their corresponding bands on the Coomassie stained gel.

*In situ* assays for Complex II (CII) (succinate dehydrogenase) proved to be relatively insensitive and irreproducible. We therefore measured this activity on mitochondria purified as described above using a method from Munujos *et al*. [40] modified for a 96 well plate format. As a negative control, an inhibitor of CII (0.5 mM thenoyltrifluoro-acetone) was added to a separate set of samples. Activity was assessed at 500 nm for 1 hour every minute on a BMG LabTech FLUOstar Omega spectrophotometer. The ΔAbs_340_/min was obtained using the maximum linear rate over a period of 20 min.

### RNA extraction and real-time qRT-PCR analysis

Total RNA was extracted from logarithmically growing cells and purified using an RNAeasy Mini kit (Qiagen, Inc., Chatsworth, CA) as previously described [23]. qRT-PCR reactions were performed with a Power SYBR Green RNA-to-CT 1-Step Kit (Life Technologies/Thermo-Fisher, Inc.) with a StepOnePlus Real-Time PCR System (Applied Biosystems, Inc. Carlsbad, CA). All primers were synthesized by International DNA Technologies, Inc. (Coralville, IA). Reactions were optimized so that single bands of the predicted size were visualized following gel electrophoresis. The real-time PCR results were calculated as relative expression after normalization to the internal standard β_2_ microglobulin using ΔΔCƬs compared to WT cells. Statistical analyses were performed using Student’s t-test. All primer sequences and amplification conditions are listed in S1 Table.

### Immunoblotting

Blotting were performed as previously described [20, 23]. All relevant antibodies used are listed in S2 Table.

### Mitochondrial oxidoreductase assays

The assay for malic dehydrogenase (MDH) relied on the conversion of oxaloacetate (OAA) to malate coupled with NADH to NAD+, which was measured using Protocol SPOXAL01 (Sigma-Aldrich). The ΔAbs_340_/min was obtained as described above.

α-ketoglutarate dehydrogenase (α-KGDH) was quantified by measuring the conversion of α-ketoglutaric acid (α-KG) to succinyl-CoA coupled to NAD+ conversion to NADH as described in Protocol SPKETO03 (Sigma-Aldrich). The ΔA_340nm_/min was calculated over 15 min using the maximum linear rate.

Isocitrate dehydrogenase (IDH) activity was quantified on 10 μg of mitochondria using an IDH Activity Assay Kit according to the directions provided by the supplier (Sigma-Aldrich). Absorbance was measured at 37C at OD_450nm_ every 5 minutes over 2 hr. with a final reading taken at the plateau stage.

Glycerol 3-phosphate dehydrogenase (G3PDH) was assayed using a G3PDH Assay Kit (Abcam, Inc., Burlingame, CA) with 10 μg of mitochondria and quantified as recommended by the manufacturer. End point absorbance (Abs_450_) was corrected for background controls.

All enzyme assays were performed on at least triplicate samples

### Enrichment and Tryptic Digestion of MEF Mitochondrial Proteins

16 MEF samples (4 each of WT, KO, WT+Myc and KO+Myc) were separately enriched for mitochondrial proteins from 10^7^ cells prepared from individual plates as described for BNGE. Protein concentrations were determined using a BCA assay (Pierce, Inc., Rockford, IL). A pooled control sample was prepared by combining equal volumes of each individual sample and was used to monitor sample preparation variation. 22 aliquots (16 samples and 6 controls) containing 20 μg of total protein were spiked with 4 μl of 125 nM ovalbumin protein. In-solution trypsin digestion was carried out as described [41]. The resulting tryptic peptides were desalted with PepClean C-18 Spin Columns (Pierce) according to the manufacturer’s protocol, vacuum-dried, and resuspended in 20 μl 0.1% formic acid.

### LC-MS/MS Analysis

Tryptic digests were analyzed using high resolution liquid chromatography tandem accuracy mass spectrometer as previously described [42]. In brief, samples were loaded with a nanoAcquity autosampler (Waters, Waltham MA) onto a capillary sample trap column, separated using a reversed phase gradient on a commercial PicoChip nanospray C18 column (PicoChip) and electrospray ionization source (New Objective, Inc. Woburn MA). Mass analysis was performed on a hybrid electrosprayed into a LTQ/Orbitrap Velos hybrid mass spectrometer (Thermo Fisher). Data dependent acquisition consisted of cycles of a high resolution full scan FT mass spectrum followed by 13 MS/MS low resolution tandem mass spectra scans in the linear ion trap, with dynamic exclusion setting enabled to minimize redundant selection of precursor ions previously selected for CID. High-resolution liquid chromatography mass spectrometry was used to measure the mass-to-charge ratio, retention time, and intensity of the isotopes for each identified peptide. Custom differential mass spectrometry software (dMS 1.0, University of Pittsburgh and InfoClinika, Seattle WA) was used to align, integrate, and link the high resolution peak areas data to the protein identification results from a COMET to the tandem MS sequence database search results [43]. In total, detection of 414,655 isotope distributions, 9,397 peptide sequences, and 1929 protein identifications were obtained from this data set.

### Selection of Mitochondrial Proteotypic Peptides

Intensities of a single representative peptide were used as surrogate markers for relative abundance of each protein. Proteotypic representative peptides were selected based upon their signal intensities and their correlation with other peptides originating from the same protein. Proteins with a single identified peptide sequence or with poor concordance among identified peptides were excluded from further analysis. For the remaining proteins, peptides with the highest signal intensities (average of all samples) among those with good correlation with other peptides from the same protein (mean Pearson’s correlation coefficient >0.5) were selected as representative peptides. A total of 345 peptides belonging to proteins annotated as having evidence of mitochondrial localization in the David (http://david.abcc.ncifcrf.gov/) Bioinformatics Database and/or mouse MitoCarta Inventory (http://www.broadinstitute.org/pubs/MitoCarta/mouse.mitocarta.html) were selected and their high resolution peak area extracted using dMS software were used for statistical analysis.

### Statistical Analysis

**Fig 1:**
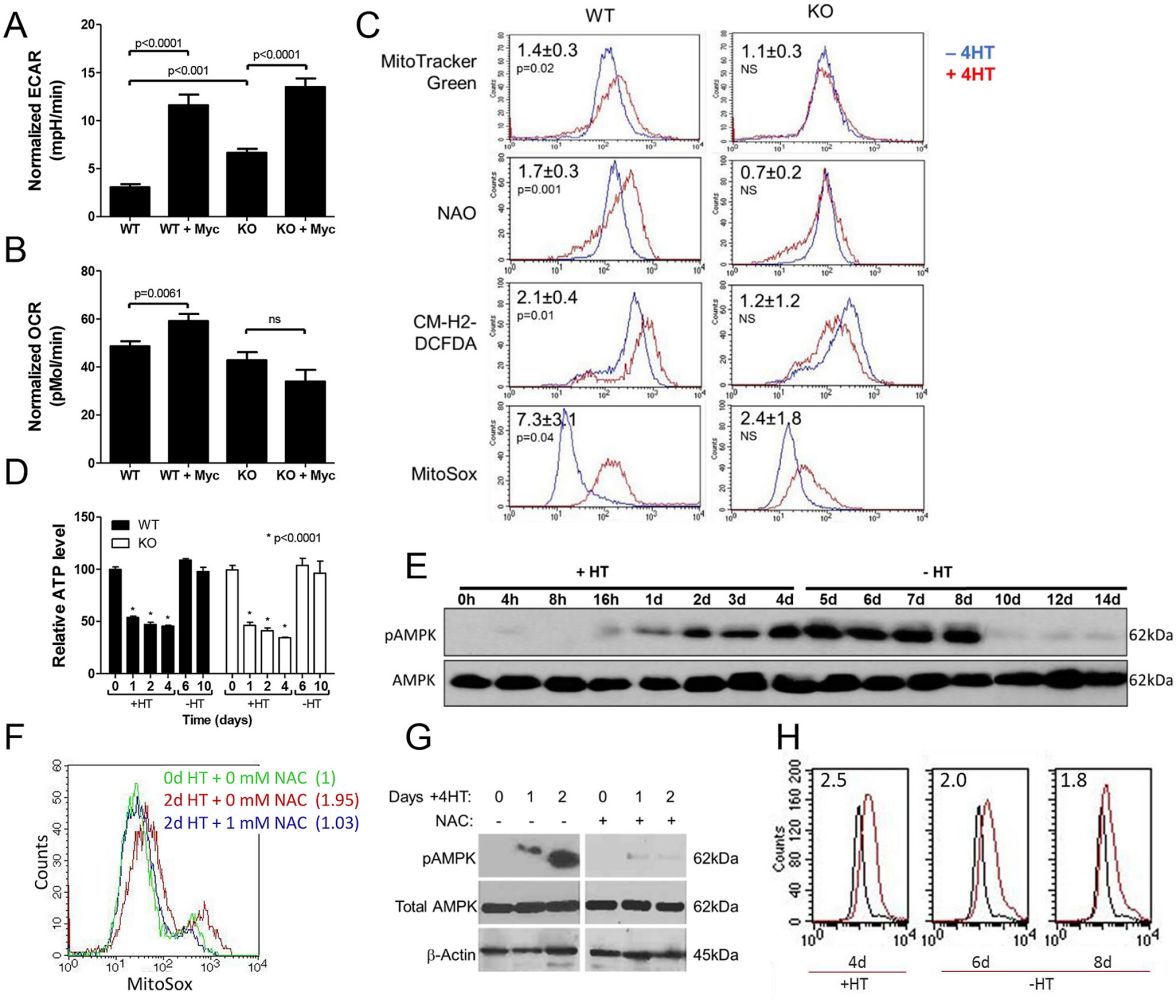
Energy-generating pathway responses to MycER activation. *(A)* Baseline glycolysis measurements normalized to cell number at conclusion of the experiment. WT and KO MEFs were either untreated or exposed to 4HT for 7 days to activate MycER (+Myc). Glycolysis was quantified by measuring extracellular acidification rates (ECAR). Bars represent the mean of quadruplicate measurements ± 1 standard error of the mean (SEM). *(B)* Baseline Oxphos measurements. Oxygen consumption rates (OCR) were simultaneously quantified on the same samples described in A. See S2 Fig for additional details regarding the glycolytic and Oxphos responses of these cells. *(C)* Changes in mitochondrial mass and ROS production. Untreated or 4HT-treated (7 days) WT and KO cells were stained with MitoTracker Green or NAO to independently assess mitochondrial mass. In parallel experiments, cells were also stained with CM-H2-DCFDA to quantify total ROS production or with MitoSOX to specifically quantify the production of mitochondrial-derived superoxide. In all cases, typical flow diagrams are depicted. The numbers in the upper left portion of each panel indicate the mean ratios of fluorescence intensities between 4HT-treated and control, untreated cells from at least 4 independent experiments ± 1 SEM *(D)* ATP levels in response to MycER activation and inactivation. ATP levels were measured at baseline (S1*B* Fig), after the indicated periods of exposure to 4HT (+) and its subsequent removal (-). Each value shown depicts the mean of quadruplicate samples ± 1 SEM. Basal (day 0) ATP levels in untreated KO cells were routinely found to be 30–40% lower than those of their WT counterparts (S1*B* Fig) but are normalized here to 100% in both cell types for easier comparison. After taking the day 0 differences into account, KO MEFs also showed significantly lower ATP levels on days +1–4 compared to their WT counterparts (P < 0.02) (S1*B* Fig). *(E)* AMPK response to MycER activation/inactivation in WT MEFs. WT cells were exposed to 4HT for the indicated times. On day 4, 4HT was removed (-4HT) and cells continued to be cultured in its absence for an additional 6 days. Total AMPK and pAMPK were assessed by immunoblotting at the time points shown. *(F)* MitoSox staining as described in Fig 1*C* after 2 days of 4HT treatment. In the concurrent presence of 1 mM NAC, no significant change in ROS was observed. (*G*) AMPK activation is suppressed by NAC. WT cells were exposed to 4HT in the absence or presence of 1 mM NAC. Total and phospho-AMPK were assessed by immuno-blotting as described for panel E. *(H)* Persistence of ROS following MycER inactivation. WT cells were exposed to 4HT for 4 days at which point ROS were quantified using MitoSox as described in *(C)*. 4HT was then removed with ROS levels again being determined 2 and 4 days later (days 6 and 8). Numbers in upper left corner indicate the ratio of mean fluorescence intensity of 4HT-treated (red curves) to non-4HT-treated MEFs (black curves).

Two way ANOVA was used to determine the influence of AMPK genotype and Myc over-expression on the abundance of mitochondrial proteins, and also to determine whether there was any significant interaction effect between AMPK genotype and Myc over-expression (i.e. whether a protein’s response to Myc was discordant between WT and KO cells). Features were selected based on a *q* value (false discovery rate) cutoff of 0.05. Myc over-expression did not affect the overall abundance of mitochondrial proteins (p values for student’s t test comparison of WT vs. WT + Myc and KO vs. KO + Myc were 0.870 and 0.761 respectively). However, we did observe a 24% higher average intensity of all mitochondrial peptides in KO samples both with and without Myc over-expression (two-way analysis of variance [ANOVA] p value of 0.002) despite the overall intensities of non-mitochondrial proteins being otherwise identical. This could be due to a slightly greater overall mitochondrial mass in KO cells although this was not confirmed by staining with NAO or MitoTracker dyes (Fig 1C). To reduce the potential effect of bias due to slightly higher amounts of mitochondrial proteins in the KO samples, proteins with greater abundance in KO but with fold change less than 2.6 (twice the fold change of overall mitochondrial abundance in KO samples) were not considered for the main effect of genotype.

### Expression of roGFP2

roGFP2 was a generous gift from Dr. Michael Palladino (The Univ. of Pittsburgh). Compared to GFP, roGFP2 contains several amino acid substitutions, including S147C and Q204C, which allow the molecule to form stable intra-molecular disulfide bonds in reduced environments [44]. Oxidation of these residues alters the tertiary structure of the protein as well as the intensity of 510 nm light emitted following alternating excitation at 410 nm and 474 nm [45].

We used standard molecular biology techniques to insert roGFP coding sequences into the pDsRed2-Mito vector (Clontech, Inc., Mountain View, CA) from which the DsRed insert had been excised. This step placed the roGFP coding sequence downstream from and in-frame with the mitochondrial targeting signal peptide from subunit VIII of the human cytochrome c oxidase ETC subunit (roGFP-mito). To generate a cytoplasmically-localized roGFP2 vector (roGFP-cyto), the roGFP-mito vector was digested with NheI and BamHI to excise the mitochondrial signal peptide and the subsequently blunt-ended vector was self-ligated. The coding region of each vector was then amplified by PCR and cloned directionally into the pLenti6/V5-TOPO lentiviral vector (Life Technologies, Inc.). After packaging in 293FT cells, WT and KO MEFs were transduced with each vector and selected in 1 μg/ml of blasticidin. Pooled blasticidin-resistant clones were then further selected by fluorescence-activated cell sorting in order to purify the brightest population, which were used for all subsequent experiments.

### Confocal microscopy and flow cytometry of roGFP-mito- and roGFP-cyto-targeted cells

WT and KO cells stably expressing roGFP-cyto and roGFP-mito were grown overnight in glass bottom 6 well plates. Fresh medium lacking or containing 4HT was then added for an additional 24 hr. Confocal images of live cells were obtained with a confocal laser scanning microscope (LSM710; Zeiss) using a 20x/0.8 M27 Plan-Apochromat objective and a 31 μm pinhole with the following excitation/emission wavelength (λex/em) settings: λex/em 405/495-575 for oxidized roGFP, λex/em 488/495–575 for reduced roGFP. To quantify changes in the cellular redox states, analyses were performed by flow cytometry on a BDFACS Aria II SORP using BD FACSDiva Software and ratios were calculated using FlowJo software. Spectra were collected using a violet laser with 405 nm excitation, emission collected using a 525/50 bandpass filter and 488 nm excitation was collected using an emission bandpass of 520/50. Control experiments to determine maximal responses to oxidized and reduced environments were performed by adding H_2_O_2_ or DTT to final concentrations of 1 mM or 10 mM, respectively for 30 min prior to flow cytometry. A third sample was treated with H_2_O_2_ for 30 min followed by the addition of DTT for an additional 30 min and then analyzed. In all cases, biological triplicates were assessed for each group and each experiment was repeated at least twice. Results were expressed as the mean ratio of oxidized: reduced fluorescent roGFP based on changes in emission spectra.

### High performance liquid chromatography-electrospray ionization tandem mass spectrometry (HPLC-ESI-MS/MS)

Analyses were conducted on a Q Exactive mass spectrometer with on-line separation by Dionex Ultimate 3000 HPLC (both from Thermo Fisher, San Jose, CA). For untargeted quantification of polar metabolites, ~10^9^ cells were extracted in 80% methanol at 0°C and then incubated at -20°C for 1 h. Thermo SIEVE (Thermo Fisher) was used for peak alignment and integration of MS results to derive the relative abundance of individual metabolites. For ATP analysis,10^9^ cells were extracted at 0°C in 15% trichloroacetic acid (TCA) containing ([^13^C_10_,^15^N_1_]ATP) as an internal standard. An aliquot of the clarified sample was then directly injected. Quantification was performed by integrating the extracted ion chromatograms of each metabolite, which were then compared with a standard curve.

For the analysis of polar metabolites, lysates from sub-confluent cell cultures were prepared as described above, clarified by centrifugation, and the supernatants were placed in autosampler vials. HPLC-ESI-MS/MS was performed on a Q Exactive mass spectrometer (Thermo Fisher) with on-line separation by Dionex Ultimate 3000 HPLC (Thermo Fisher). HPLC was performed as described by Paredes *et al*. [46], with some modification: column, Luna NH_2_, 3 μm, 2 x 150 mm (Phenomenex, Inc., Torrance, CA); mobile phase A, 5% acetonitrile in water with 20 mM ammonium acetate and 20 mM ammonium hydroxide, pH 9.45; mobile phase B, acetonitrile; flow rate, 300 μL/min; gradient, 85%-1% B over 10 minutes and held at 1% B for 10 minutes. Full scan mass spectra were acquired in the orbitrap using negative ion detection over a range of m/z 100–800 at 70,000 resolution (m/z 300). Metabolite identification was based on the metabolite accurate mass (± 5 ppm), manual interpretation of the MS/MS fragment patterns, and agreement with the HPLC retention time of authentic standards. Thermo SIEVE was again used to process raw data files in order to quantify metabolites of interest. Peak alignment and integration were performed and relative abundances of each metabolite were generated among different samples.

Nucleotides were quantified from cell lysates prepared as described above. Cell pellets were extracted at 4°C with 15% TCA containing ^13^C,^15^N-ATP as the internal standard, and then neutralized with a mixture of trioctylamine and 1,1,2-trichlorotrifluoroethane. LC-MS analyses were performed on a Q Exactive mass spectrometer with on-line separation using Dionex Ultimate 3000 HPLC (both from Thermo Fisher). HPLC conditions similar to those detailed in Zhou *et al*. [47], with some exceptions: Waters XTerra-MS C18 column (3.5 μm, 2.1 x 150mm); mobile phase A, 5 mM hexylamine and 0.5% diethylamine in water, pH10; mobile phase B, 50% acetonitrile in water; flow rate, 400 μL/min; gradient, 1%-20% B over 10 minutes and followed by 20%-30%B over 5 minutes. Full scan mass spectra were acquired in the orbitrap using negative ion detection over a range of m/z 300–800 at 70,000 resolution (m/z 300). Identification of metabolites was based on the metabolite accurate mass (± 5 ppm) and agreement with HPLC retention times of standards. Quantification was achieved by integrating the extracted ion chromatograms of individual metabolites and compared with the appropriate standard curves.

### Pyruvate dehydrogenase (PDH), pyruvate kinase (PK) assays, and acetyl CoA assays

PDH activity was quantified using the PDH Enzyme Activity Microplate Assay Kit according to the directions provided by the supplier (MitoSciences, Eugene, OR). Triplicate samples were loaded at 1 mg/well, measured kinetically over 30 min and the rates were determined as changes in OD (ΔOD) over time.

PK activity was determined using a modified protocol from Worthington Biochemical Corp (Lakewood, NJ) in which the conversion of phospho(enol)pyruvate (PEP) to pyruvate by PK is coupled to the conversion of lactate to pyruvate and the generation of NAD+. 1 plate of semi-confluent cells was trypsinized and resuspended in 50 mM imidazole HCl buffer, pH 7.6 containing 12 mM KCl and 62 mM MgSO4 at 10^6^ cells/ml. 100 μl of cells were dispensed into individual wells of a 96 well plate containing a reaction solution whose final components consisted of 1.36 mM ADP, 1.36 mM PEP, 0.4 mM NADH and 40–45 U of lactate dehydrogenase in imidazole buffer to a final volume of 200 μl. NADH conversion to NAD+ was measured at 340 nm for 15 min and the rate was determined by ΔAbs_340_/min from quadruplicate reactions.

Acetyl CoA was assayed using an Acetyl Coenzyme A assay kit and was performed as recommended by the supplier (Sigma-Aldrich). Samples were prepared as described by Edmunds *et al*. [23]. Triplicate samples were compared to a 1 nmol standard curve using a SpectraMax M2 fluorescence plate reader and analyzed by Students’s t-test.

## Results

### AMPK is necessary for Myc-stimulated mitochondrial biogenesis and function

To investigate the role for AMPK in mediating mitochondrial structure and function in the basal state and in response to Myc activation, we stably expressed the MycER fusion protein [37] in immortalized *ampk+/+* (WT) and *ampk-/-* (KO) MEFs (S1*A* Fig) [34, 35]. In both cases, the cells were of identical size and grew at similar rates (not shown). In response to the estrogen analog 4-hydroxytamoxifen (4HT), both cell types increased their rates of glycolysis although basal levels were higher in KO cells (Fig 1*A* *and* S2*A* & S2*B* Fig). Cells had very little glycolytic reserve when simulated by oligomycin (S2*A* and S2*B* Fig), implying that all lines were operating at near maximal glycolytic capacity. In contrast, KO cells failed to up-regulate Oxphos in response to MycER activation (Fig 1*B* *and* S2*C* & S2*D* Fig). Thus, at least in MEFs, basal levels of glycolysis and Myc’s up-regulation of Oxphos are AMPK-dependent. Very little oxygen was consumed for non-mitochondrial respiration in any of the cell lines (S2*C* and S2*D* Fig), indicating that no respiration was occurring as a result of side reactions attributable to factors such as peroxisomal oxidation. The trends of Oxphos and glycolysis recorded for basal respiration (Fig 1*A* and 1*B*) continued throughout the mitochondrial stress test (S2 Fig).

To determine whether KO cells’ failure to increase Oxphos in response to MycER activation was indicative of a more global degree of mitochondrial unresponsiveness, we quantified changes in mitochondrial mass and production of reactive oxygen species (ROS), both of which are normally increased by Myc over-expression [20, 21, 48]. MycER activation in WT cells was accompanied by a reproducible 20–30% increase in mitochondrial mass as evidenced by staining with both MitoTracker Green and Acridine orange 10-nonyl bromide (NAO) as well as by an increase in ROS [20, 23] (Fig 1*C*). The likely mitochondrial origin of ROS was also confirmed by MitoSox staining, which detects superoxide (O_2_^-^). The marked attenuation or absence of these responses in KO cells indicated that, in addition to Oxphos, other Myc-regulated mitochondrial responses are also AMPK-dependent.

We assessed the bio-energetic consequences of the above differences by measuring ATP levels in WT and KO MEFs during the course of MycER activation and subsequent inactivation. Basal (day 0) ATP levels in KO MEFs were routinely 30–40% lower than those in WT MEFs (S1*B* Fig). In both cases, ATP levels declined within 24 hr. of MycER activation, remained low throughout the ensuing 4 days and then normalized within 48 hr. of 4HT removal. However, the degree of ATP depletion in KO MEFs was greater than in WT MEFs, particularly after accounting for the lower basal levels in the former cells (Fig 1*D* and S1*B Fig*). Concurrent immuno-blotting performed on WT cells showed an inverse relationship between AMPK activation and ATP levels (Fig 1*E*) as well as the persistence of AMPK activation for at least 4 additional days beyond the time at which ATP levels had returned to pre-treatment levels. This suggested that factors other than adenosine nucleotide levels might also be contributing to AMPK activation. Because ROS can also activate AMPK [49–52], we repeated the above experiment in the presence of the anti-oxidant N-acetylcysteine (NAC) at a concentration that reduced ROS in 4HT treated cells (Fig 1*F*). Under these conditions, AMPK phosphorylation was markedly attenuated (Fig 1*G*). AMPK’s activation beyond the point of ATP normalization also correlated with the persistence of high ROS following 4HT removal (Fig 1*H*). Thus, in the face of Myc deregulation in WT cells, the prolonged activation of AMPK is likely to be a consequence of both ATP depletion and the persistence of ROS.

**Fig 2:**
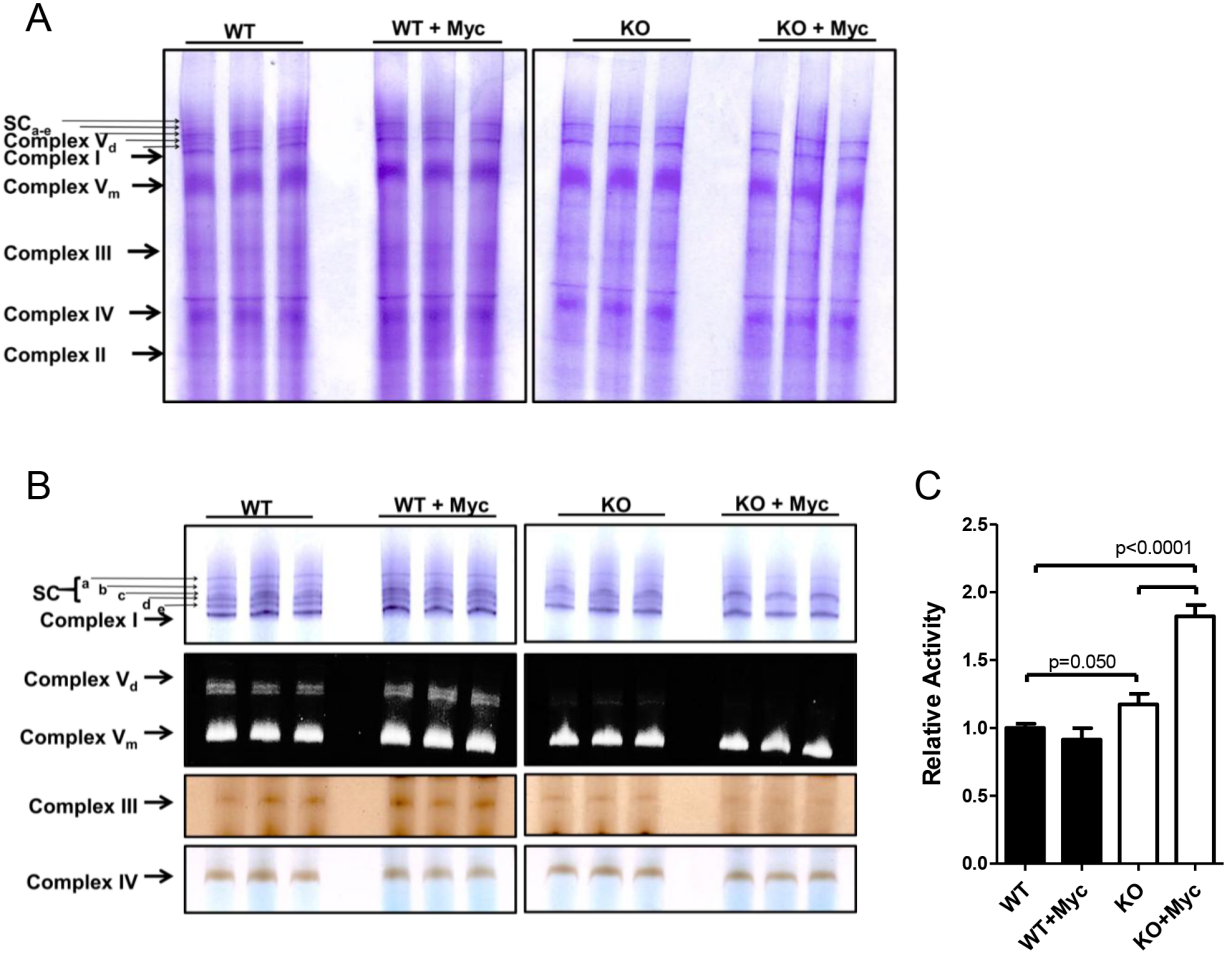
Structural and functional properties of ETCs complexes in WT and KO cells. *(A)* Analysis of ETC complexes by non-denaturing BNGE. Mitochondria were purified from triplicate cultures of the indicated cell types and resolved by BNGE. At least 5 individual supercomplexes (SC_a-e_) could be resolved [20]. Note that Complex V (ATP synthase) is composed of both monomers (V_m_) and dimers (V_d_). *(B) In situ* enzymatic assays for Complex I (NADH ubiquinone oxidoreductase) plus supercomplexes, Complex V_m_ and V_d_ (ATPase), Complex III (decylubiquinol cytochrome c oxidoreductase) and Complex IV (Cytochrome c oxidase). Each assay was performed in triplicate on at least 2 occasions, with representative results being shown here (also see S2 Fig). *(C)* Complex II (succinate dehydrogenase) activity was quantified on lysates prepared from purified mitochondria as its *in situ* assessment was found to be unreliable. The results shown in the histogram represent the mean of triplicate enzymatic determinations ± 1 SEM. See S3 Fig for quantification of all enzymatic measurements.

ETC structure and function are highly Myc-responsive in *myc-/-* rat fibroblasts [20]. To determine whether these properties were also AMPK-dependent in MEFs, we used blue native gel electrophoresis (BNGE) to evaluate the integrity of each of the 4 multi-protein components of the ETC (Complexes I-IV) along with the monomeric and dimeric forms of Complex V (ATP synthase) termed V_m_ and V_d_, respectively. BNGE can also resolve ETC “supercomplexes” (SCs), which are comprised primarily of higher order associations of Complexes I, III and IV in varying stoichiometries and are believed to promote more efficient ETC function [53, 54]. Individual BNGE components of Complexes I-V from WT and KO MEFs were similar in appearance and did not change appreciably following Myc induction (Fig 2*A*). In contrast, at least 5 SCs (SC_a_-SC_e_) and Complex V_d_, readily seen in WT MEFs, were reduced or absent in KO cells (Fig 2*A*).

Activity assays for individual complexes better emphasized the differences between WT and KO MEFs both prior to and following MycER activation (Fig 2*B* and 2*C* & S3 Fig). For example, neither Complex I nor Complex V_m_ activity was significantly altered under either condition whereas Complex V_d_ was virtually absent in KO cells regardless of MycER status. In contrast, Complex III activity was decreased in KO cells regardless of MycER status whereas Complex IV decreased only in response to MycER activation. Consistent differences were also observed in the enzymatic activities of several SC components (Fig 2*B* and S3 Fig). Specifically, SC_a_ activity was lower in KO cells, irrespective of Myc’s activation status, whereas changes in the remaining SCs were detected only in KO cells following MycER activation (S3 Fig). Complex II activity was slightly higher in KO cells but increased significantly following MycER activation whereas no change was evident in WT cells (Fig 2 *C*). Thus, while MycER activation had little to no discernible effect on the ETC enzymatic activity of WT cells, it had a pronounced effect in KO cells. This provided further evidence that AMPK is needed to maintain normal mitochondrial function both in the basal state and in response to Myc deregulation.

### Transcriptional and enzymatic profiling reveals co-operativity between Myc and AMPK in modulating metabolic function

**Fig 3:**
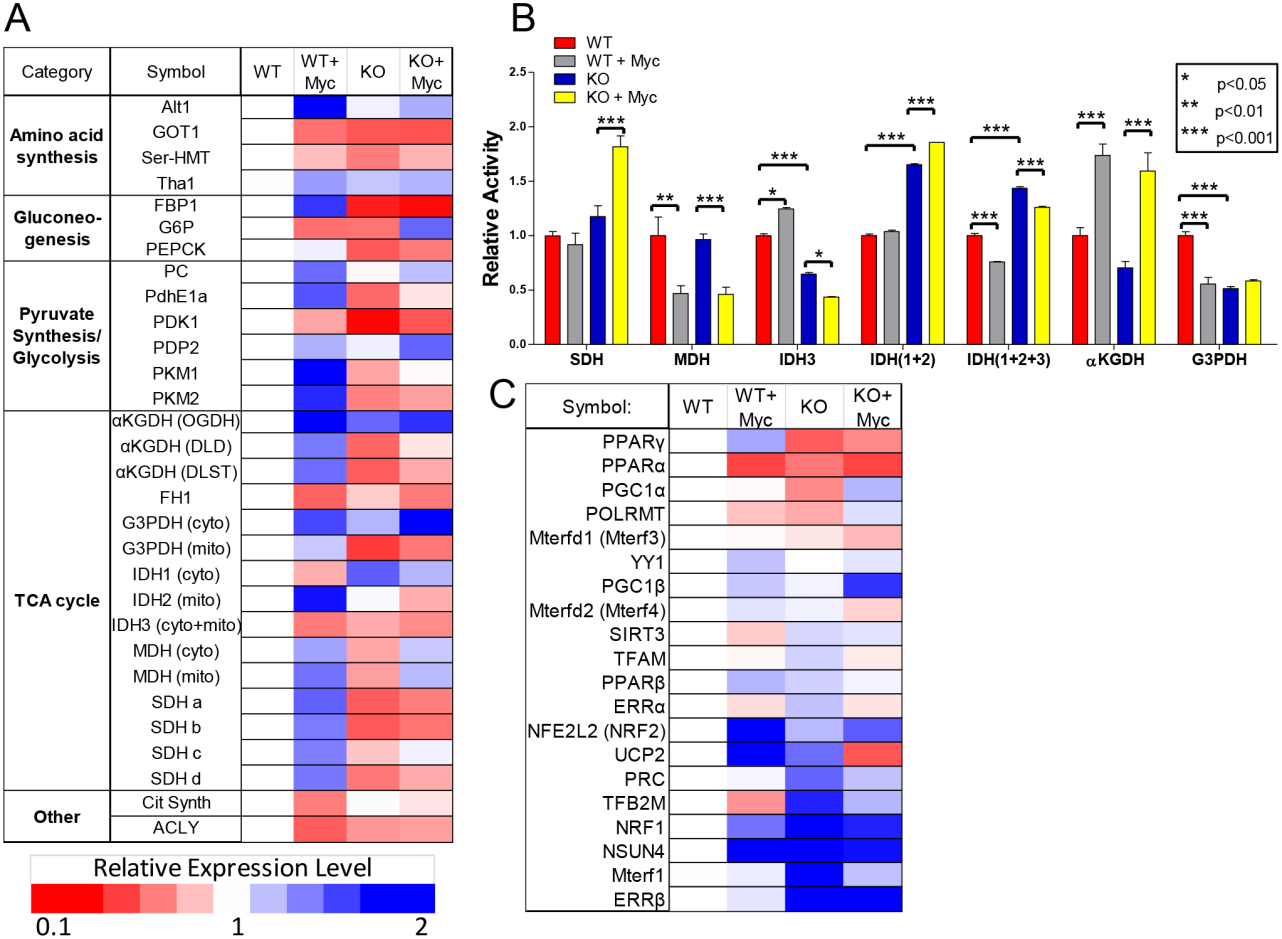
Transcriptional and enzymatic differences between WT and KO MEFs. (A) Transcriptional profiling. Real-time qRT-PCR was performed in triplicate for each of the indicated transcripts. The levels of each transcript determined in WT cells were arbitrarily set to 1 as indicated by the white boxes. See S4 Fig for the actual numerical values and p values for individual determinations. (B) Functional assays for oxidoreductases. Each bar represents the mean of biological triplicate determinations ± 1 SEM performed on purified mitochondrial lysates. Note that the results for SDH are the same as those presented in Fig 2C. (C) qRT-PCR profiling of transcripts involved in the regulation of mitochondrial DNA replication and maintenance, transcription and function. Analyses were performed as described for (A). See S5 Fig for the actual numerical differences and p values for individual determinations.

Given AMPK’s influence on both basal and Myc-dependent mitochondrial functions, we performed a small-scale gene expression survey of WT and KO cells. We examined 30 transcripts whose encoded proteins comprise key elements of glycolysis, the TCA cycle and other pathways relevant to energy regulation and mitochondrial function. Significant differences were noted between WT and KO MEFs with 19 transcripts being relatively under-expressed in the latter cells, 3 over-expressed and 8 unchanged (Fig 3*A* and S4 Fig). Following MycER activation, WT cells showed significant up-regulation of 19 transcripts and down-regulation of 9, in a pattern that generally correlated with the increased rates of glycolysis and Oxphos (Fig 1*A* and 1*B*). In contrast, the transcriptional response to MycER activation in KO cells was markedly different with only 12 transcripts demonstrating any significant change (Fisher’s Exact test, P < 0.0001). Moreover, 19 of the 28 Myc-responsive transcripts in WT cells were expressed discordantly in KO cells. Particularly noteworthy examples included transcripts for glucose 6-phophatase (G6P); the M2 isoform of pyruvate kinase (PKM2); isocitrate dehydrogenase 2 (IDH2) and each of the 4 subunits of succinate dehydrogenase (SDHa-d), which comprise Complex II of the ETC. Collectively, these studies indicate that AMPK functions to coordinate the transcriptional activity of the majority of transcripts examined herein both in their basal state and in response to MycER activation.

The actual enzymatic activities of several mitochondrial oxidoreductases encoded by the above transcripts were also discordant (Fig 3B). For example, basal activity of NADH-dependent isocitrate dehydrogenase (IDH) was higher in WT cells and increased in response to Myc activation whereas it declined in KO cells. A different pattern was observed in the case of glycerol 3-phosphate dehydrogenase (G3PDH), which was lower in basal-state KO cells than in WT cells; it also failed to respond to Myc over-expression in contrast to WT cells where its activity declined. That not all enzymatic activities were altered as a consequence of the loss of AMPK was evidenced by malate dehydrogenase (MDH) levels, which were identical in basal-state WT and KO cells and decreased commensurately in response to Myc over-expression. As is commonly the case for many transcripts and their encoded proteins and for individual subunits of multi-protein complexes [55–61] the measured enzyme activities did not closely correlate with the relevant transcript levels shown in Fig 3A. Collectively, these findings are consistent with those depicted in Fig 2 and S3 Fig and support the notion that some TCA cycle enzymes rely upon AMPK either for their basal function or their proper response to Myc over-expression.

The above studies suggested that both AMPK and Myc might be controlling more proximal determinants of Oxphos. We therefore conducted a qRT-PCR-based survey for 20 known regulators of mitochondrial structure and function including those involved in DNA replication and transcriptional maintenance [62–65]. 13 of the tested transcripts were found to be differentially expressed between WT and KO cells, with 10 up-regulated in KO cells and 3 down-regulated (Fig 3C and S5 Fig). Following MycER activation, significant additional differences were noted. For example, in contrast to the up-regulation of 6 transcripts and the down regulation of 2 transcripts in WT cells in response to MycER activation, KO cells responded anomalously, with only 3 transcripts up-regulated and 9 down-regulated. Within these subsets of Myc-responsive transcripts, the largest differences in WT cells included a 2.4-fold increase in NSUN4 and a 4.6-fold increase in UCP2 whereas in KO cells, Mterf1 and UCP2 transcripts were reduced by 3.4- and 3.9-fold, respectively. These results indicate that AMPK and Myc cooperate to ensure the proper coordination of numerous factors that supervise mitochondrial-specific DNA replication and transcription.

### Differences in mitochondrial proteomes of WT and KO MEFs

Aiming to identify changes in the relative abundance of mitochondrial proteins between WT and KO MEFS both prior to and in response to de-regulated Myc over-expression, we applied a differential mass spectrometry (dMS) workflow to a set of enriched mitochondrial samples isolated from WT and KO cells either prior to or following an 8 day period of Myc activation [66, 67]. We reliably identified and quantified the relative abundance of 345 mitochondrial proteins as annotated in the DAVID Bioinformatic Database and/or the Mouse MitoCarta Inventory of Mammalian Mitochondrial Genes (S3 Table). High-resolution liquid-chromatography Fourier transformed mass spectrometry was used to detect these proteins by comparing the relative intensity of individual high-resolution isotope distributions across each of the analyzed samples (S6 Fig). We observed a 24% higher average intensity of all mitochondrial peptides in KO samples both with and without Myc over-expression 2-way analysis of variance (ANOVA) p value of 0.002] compared to WT samples, despite the overall abundance of non-mitochondrial proteins being otherwise identical. This may be caused by a slightly higher purity of mitochondria in the former samples. It could also be due to a slightly greater overall mitochondrial mass in KO cells, although this was not observed by staining with NAO or MitoTracker dyes (Fig 1*C*).

**Fig 4:**
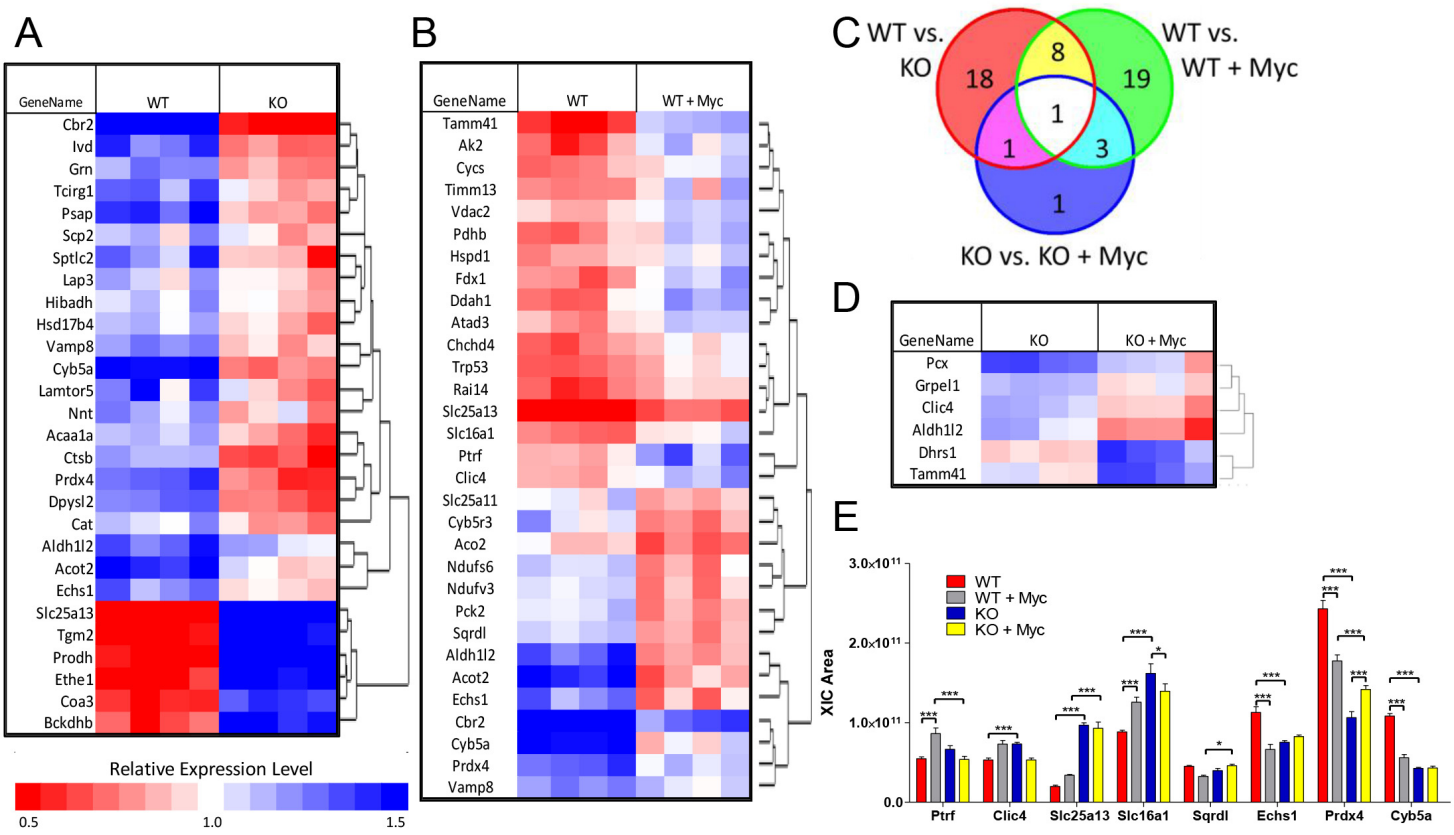
Mitochondrial proteomic profiling. LC-MS/MS reliably identified and quantified 345 proteins with mitochondrial localization from representative peptides in each of the 4 experimental groups (S3 Table). (A) Differential protein expression between WT and KO MEFs prior to MycER activation. (B) Differential protein expression in WT MEFs prior to or following an 8 day period of MycER activation. (C) Venn diagram of protein overlap between WT and KO MEFs, WT vs. WT+Myc and KO versus KO+Myc. Each of the protein subsets within these 3 groups are enclosed by red, green and blue circles and correspond to the proteins denoted in panels A, B and D, respectively. All proteins were selected by a conservative q-value of <0.05 by 2-way analysis of variance (two way ANOVA). (D) Differential protein expression in KO MEFs prior to or following 8 days of Myc over-expression. (E) Quantification of 8 proteins that showed a significant interaction effect by both AMPK and Myc overexpression.

Using a conservative q-value cutoff of 0.05, we determined the abundance of 28 proteins to be significantly different between WT and KO MEFs prior to MycER activation with 22 being more abundant in WT cells and 6 more abundant in KO cells (Fig 4*A* and 4*C*, red circle). Following 8 days of MycER activation, 31 mitochondrial proteins were altered in WT cells, with 17 up-regulated and 14 down-regulated (Fig 4*B* and 4*C*, green circle). 8 of these (26%) were also members of the group depicted in Fig 4*A* that were thus also AMPK-responsive (Fig 4*C*, yellow). In contrast, only 6 proteins were found to be Myc-responsive in KO MEFS (Fig 4*D* and 4*C*, blue circle). Of these, Tamm41, Aldh1l2, and Clic4 were also influenced by Myc in WT cells (Fig 4*C*, cyan). The response of 8 proteins to Myc over-expression was also found to be discordant and having a significant “interaction effect” (Fig 4*E*), with 5 of them (Prdx4, Sqrdl, Slc16a1, Clic4 and Ptrf) changing their abundance in opposite directions in WT vs. KO cells, and the other 3 (Cyb5a, Echs1, and Slc25a13) only being affected by Myc over-expression in WT cells. Together, these findings indicate that approximately 15% of evaluable mitochondrial proteins (i.e. 51 of 345) can be conservatively described as being regulated by AMPK and/or Myc and that much of the long-term mitochondrial proteomic adaptation to Myc deregulation is AMPK-dependent.

### Differential redox states of WT and KO cells

**Fig 5:**
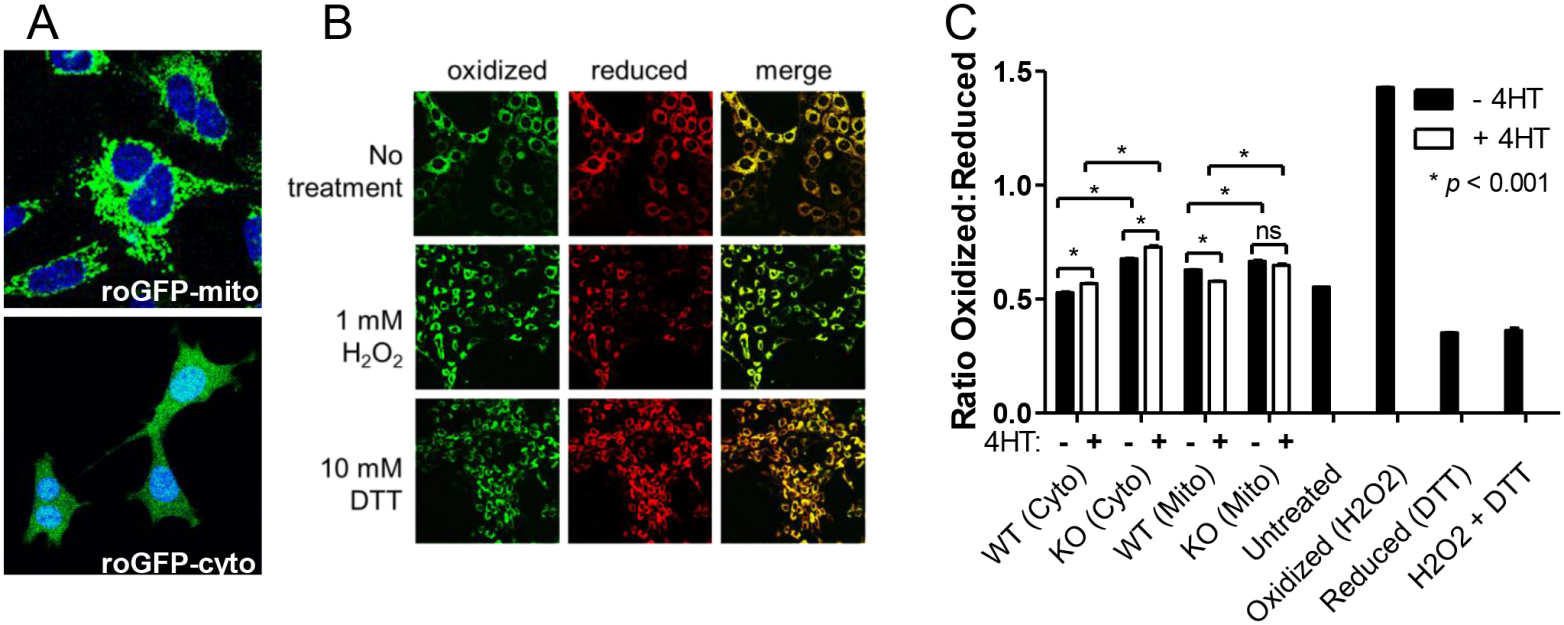
Redox states in cytoplasmic and mitochondrial compartments of WT and KO MEFs. *(A)* Live cell confocal images of WT MEFs stably expressing roGFP-mito and roGFP-cyto demonstrating specific mitochondrial and cytoplasmic localization, respectively. Nuclear counter-staining was with Hoechst 3334. *(B)* Live cell confocal microscopy of KO MEFs expressing roGFP-mito. Cells were untreated or exposed to 1 mM H_2_O_2_ or 10 mM DTT for 30 min prior to obtaining images. *(C)* Quantification of redox differences between WT and KO MEFs. Monolayer cultures were grown for 24 hr. in the absence or presence of 4HT. Flow cytometry was then used to quantify the mean fluorescence ratios of oxidized and reduced roGFP. Each bar represents the average ± 1 SEM of mean fluorescence intensities obtained from 3 independent plates of cells. * = P<0.001. Similar results were independently obtained in 2 repeat experiments as well as in 2 experiments performed following a longer period of MycER activation (7 days, not shown). The 4 bars on the right represent control experiments in which WT cells expressing roGFP-cyto were exposed under the conditions described in *(B)*. These values define the maximal possible degree of oxidation or reduction capable of being achieved under the most extreme conditions.

The differential activities of the ETC, mitochondrial dehydrogenases and the glycerol phosphate shunt (represented by G3PDH), as well as altered ROS production, might be expected to exert distinct effects on the redox states of WT and KO cells [68–71]. We tested this directly by stably targeting a redox-sensitive form of green fluorescent protein (roGFP2) [44, 72] to either the cytoplasm or mitochondrial matrix of WT or KO cells (Fig 5*A*). In control experiments, exposure of roGFP-expressing WT or KO cells to H_2_O_2_ markedly increased the ratio of oxidized:reduced roGFP, irrespective of its subcellular location (i.e. roGFP-cyto versus roGFP-mito) whereas treatment of the same cells with DTT alone or with H_2_O_2_ followed by DTT shifted the ratio to that of a more reduced state (Fig 5*B* and 5*C* and data not shown). The responses of WT and KO cells to these extreme stresses were indistinguishable (not shown) and defined the limits within which supra-physiologic changes in the redox state can occur.

Quantification of oxidized and reduced roGFP2 in the mitochondrial and cytoplasm of WT and KO cells prior to or following MycER activation for 24 hr. showed both compartments to be relatively reduced (Fig 5*C*) and in agreement with previous findings in other cell types [44, 45, 73, 74] However, in the basal state, KO cells showed a 28% greater degree of cytoplasmic oxidation (p<0.001) and a 6% greater degree of mitochondrial oxidation than WT cells (p<0.001). MycER activation in WT cells led to a further 9% increase in cytoplasmic oxidation (p = 0.0004) and an 8% increase in mitochondrial reduction (p<0.0001). Finally, although an identical 9% increase in oxidation occurred in the cytoplasm of KO cells following MycER activation (p = 0.002), no change occurred in their mitochondrial redox state (p = 0.2). These results are consistent with those of our preceding studies indicating that AMPK and Myc cooperate to promote mitochondrial biogenesis and function. They further indicate that mitochondrial structural and functional alterations mediated by Myc de-regulation are associated with significant differences in the redox state of the mitochondrial matrix.

### AMPK influences Myc-mediated re-programming of steady-state metabolites

**Fig 6:**
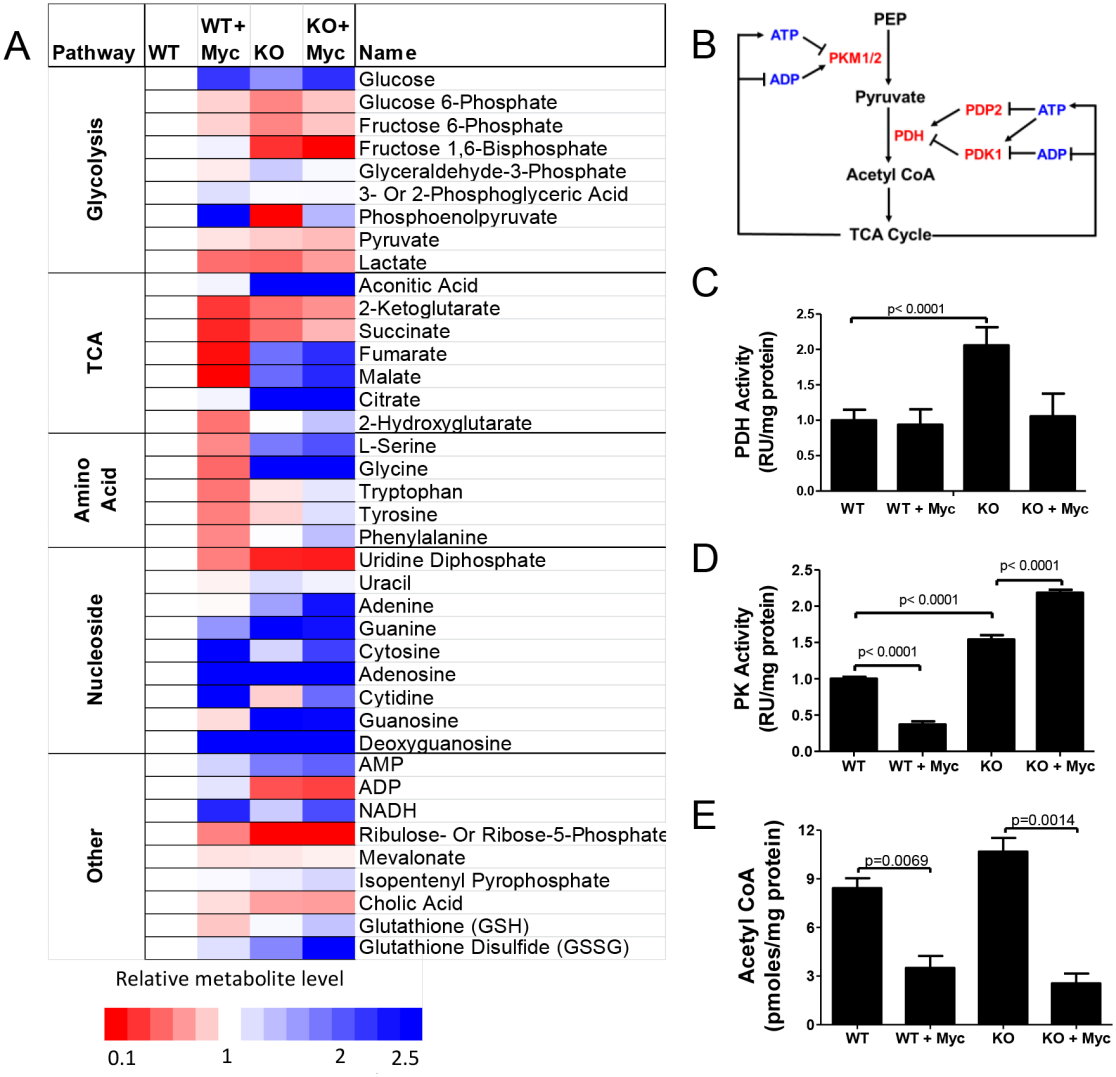
Metabolite profiling of WT and KO MEFs. (A) HPLC-ESI-MS/MS quantification of select metabolites in MEFs prior to or following MycER activation for 8 days. Each box represents the average of biological quadruplicate samples. Levels of each depicted metabolite were arbitrarily set to 1 in WT cells (white boxes). (B) Enzymatic and metabolite feedback control of PDH and PK. Note the control of the former by the stimulatory phosphatase PDP2 and the inhibitory kinase PDK1 as well as additional indirect and direct control of PDH and PK, respectively by ATP and ADP. PDH and PK enzyme activities (C and D, respectively) and acetyl CoA assays (E) were performed on whole cell extracts as previously described [23].

We used high performance liquid chromatography-electrospray ionization tandem mass spectrometry (HPLC-ESI-MS/MS) to quantify steady state levels of a select group of metabolites in WT and KO cells prior to or following MycER activation. The metabolites were chosen to reflect relevant glycolytic and TCA cycle intermediates, anabolic substrates and determinants of cellular redox and energy status. Significant differences were seen between WT and KO cells prior to MycER activation with the most striking being the generally lower levels of glycolytic substrates and higher levels of TCA cycle substrates in KO cells (Fig 6*A*). This suggested that the higher glycolytic rate of KO cells (Fig 1*A*) might be responsible for depleting some of the intermediates in this pathway whereas defects in the ETC might allow a buildup of TCA intermediates. Higher levels of nucleosides and deoxynucleosides in KO cells were consistent with the previously report of AMPK being a negative regulator of the Warburg effect [75], which could also explain the relative depletion of glycolytic substrates if they were being shunted into Warburg-related pathways. The modestly higher levels of AMP and ADP in WT cells in response to MycER activation, together with reduced ATP levels (Fig 1D and S1B Fig), is likely sufficient to account for any activation of AMPK that is not otherwise attributable to ROS (Fig 1E). In contrast, the even higher levels of AMP in KO cells, both prior to and following MycER activation, can likely be explained by the inability to normalize ATP and AMP in AMPK’s absence.

MycER activation was also associated with other distinct metabolite re-distribution patterns. Most notably WT cells showed an overall reduction of TCA cycle intermediates that most likely reflected their rapid consumption in response to increased mitochondrial mass and metabolic activity (Fig 1B and 1C) and/or a reduction of acetyl CoA entering the TCA pathway, perhaps as a result of the Warburg effect as noted above. In contrast, changes in TCA cycle intermediate levels were less pronounced in KO cells following MycER activation, which was again consistent with their generalized mitochondrial unresponsiveness (Fig 1A and 1C). Despite differences in the basal glycolytic intermediate content of WT and KO cells, the redistribution of these molecules was somewhat similar in response to MycER activation, with the exception of fructose-1,6-bisphospate. For example, both cell types showed significant increases in intracellular glucose content, likely reflecting the known propensity for Myc to increase glucose transport [5, 16]. Phosphoenolpyruvate (PEP) was also markedly increased in both cell lines (Fig 6*A*). Other notable differences included a generalized decrease in the free amino acid content of WT cells in contrast to either no change or an increase in KO cells. Both cell types also up-regulated nucleoside and deoxynucleoside pools in response to MycER activation. Finally, and consistent with their more highly oxidized cytoplasm (Fig 5C), KO cells contained higher levels of glutathione disulfide (GSSG), which is the oxidized form of glutathione (GSH) [76]. Moreover, both cell types increased their GSSG:GSH ratio in response to Myc activation in a manner that closely mirrored the accompanying changes in cytoplasmic roGFP fluorescence (Fig 5C).

To investigate potential mechanism(s) underlying some of the differences in metabolite distribution between WT and KO cells, we next examined pyruvate dehydrogenase (PDH) and pyruvate kinase (PK). These enzymes are notable for catalyzing 2 of the 3 irreversible steps in glycolysis (ΔG = -7.5kcal each) and are subject to complex and multi-factorial positive and negative regulation (Fig 6*B*) [77–79]. For example, in addition to modifications such as acetylation and oxidation and feedback regulation by adenosine nucleotides, PDH is also tightly controlled post-translationally by the inhibitory Ser/Thr kinase pyruvate dehydrogenase 1 (PDK1) and the stimulatory phosphatase pyruvate dehydrogenase phosphatase 2 (PDP2) both of which regulate the level of Ser_293_ phosphorylation of the PDH E1 subunit (PDHE) [80, 81]. PK activity is typically regulated by changes in the abundance of its PKM1 and PKM2 isoforms, with the expression of the latter tending to correlate with high rates of proliferation [77, 78]. This, together with the significantly higher K_m_ of PKM2 is believed to account for the accumulation of substrates upstream of PEP that drive the Warburg effect and limit the availability of glycolytically-derived acetyl CoA for usage by the TCA cycle [78, 82]. Additionally, both PK isoforms are also subject to multiple and non-mutually exclusive types of post-translational modification; allosteric feedback by metabolites such as ATP, ADP and fructose-1,6,-bi-phosphate; and changes in enzyme dimer: tetramer ratios [83, 84].

Immunoblotting of WT and KO cell extracts both prior to and after MycER activation showed minimal changes in protein levels of PDHE, PKM1 and PKM2 (S7 Fig). A reduced amount of pPDHE1 was observed in KO cells although it did not change appreciably in response to MycER activation. Decreases in PDK1 and increases in PDP2 occurred in WT and KO cells following MycER activation.

Changes in the enzymatic activities of PDH and PK were more revealing. As seen in Fig 6C, PDH activity was significantly higher in KO cells but only in the basal state. PK activity was also higher in KO cells, and increased even further in in KO cells in response to MycER activation, but decreased in WT cells after MycER activation (Fig 6D). Together, these results are consistent with the idea that Myc and AMPK cooperatively regulate the switch between glycolysis and Oxphos by coordinating the activities of two critical and irreversible glycolytic steps that are important for determining the availability of acetyl CoA and its use by the TCA cycle. Interestingly, despite the above-noted differences in PDH and PK activities between WT and KO cells, the absolute abundance of acetyl CoA, either in the basal state or in response to MycER activation was quite similar in WT and KO cells (Fig 6*E*).

## Discussion

### Mitochondrial responses to Myc over-expression are AMPK-dependent

**Fig 7:**
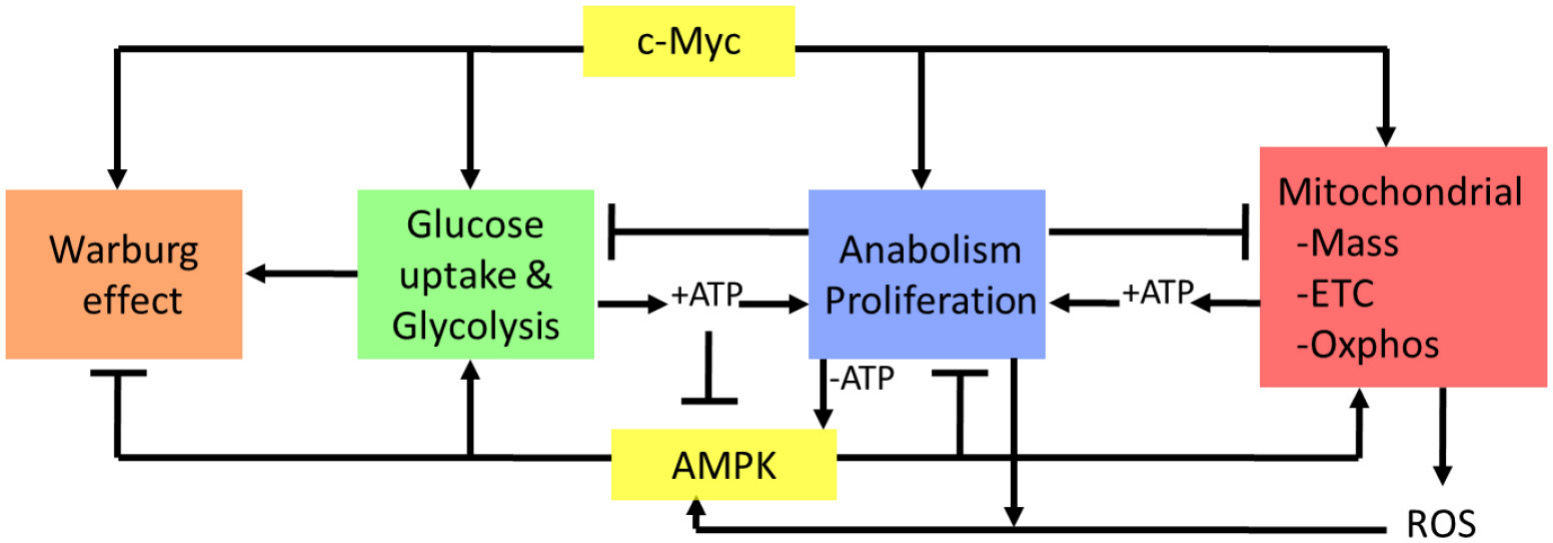
Model depicting the relationship between Myc and AMPK (yellow boxes) demonstrating their influence over common metabolic functions, although sometimes in opposite ways and for different purposes. Communication between Myc and AMPK may occur via at least 2 distinct and semi-autonomous routes with different initiating events and consequences. The first involves the activation of AMPK via Myc-mediated depletion of cellular ATP stores arising as a consequence of energy-consuming anabolic processes such as proliferation (-ATP, blue box) (Fig 1D and [23]). The second involves AMPK activation via ROS generated from increases in mitochondrial metabolism or cytoplasmic signaling pathways. It is notable that, in the first case, AMPK activation is dependent upon ATP depletion whereas, in the second case, AMPK activation occurs regardless of ATP status. AMPK activation via ROS can thus anticipate impending ATP depletion and prevent or limit this by down-regulating ATP dependent processes. In the face of a pre-existing ATP deficit, other functions of AMPK such as the promotion of proliferative arrest might tend to override the effects of Myc over-expression. In contrast, activation of AMPK by ROS might reinforce an already highly proliferative and ATP-replete state by promoting pro-anabolic functions such as glycolysis and Oxphos without necessarily compromising proliferation.

Considerable evidence supports the idea that Myc and AMPK influence similar cellular and biochemical functions although via different pathways and often for different purposes (Fig 7). For example, both promote glucose uptake, glycolysis, mitochondrial biogenesis and Oxphos [5, 20, 21, 27–29, 32, 33]. In response to Myc over-expression, these activities are believed to provide the crucial anabolic precursors and ATP needed to support the highly energy-consuming process of biomass accumulation [16, 19]. In fact, much of the increased glycolytic flux mediated by Myc over-expression is currently viewed as being directed towards promoting the Warburg effect and anabolism while being diverted away from mitochondrial consumption (Fig 7 and [5, 18, 85]). Similarly, TCA cycle intermediates, some of which originate from extra-glycolytic sources such as glutaminolysis and fatty acid β-oxidation, may also be directed into non-mitochondrial biosynthetic pathways in a Myc-dependent manner [5, 22, 23]. Moreover, Myc’s enhancement of ETC activity increases ATP production and its rate of turnover to support anabolism and proliferation [20]. In contrast, AMPK activation occurs in response to critical energy shortages, which suppress energy-consuming processes while activating energy-generating ones [27]. Thus AMPK activation opposes the Warburg effect thereby reducing the flow of glycolytic substrates into anabolic pathways and maximizing their eventual conversion into acetyl CoA for utilization by the TCA cycle [27, 75]. The cross talk between Myc and AMPK is readily apparent in *myc-/-* fibroblasts, which constitutively express high levels of phosphorylated AMPK as a consequence of their being unable to restore their ATP deficit by up-regulating glycolysis or Oxphos [23].

The failure of KO cells to increase Oxphos in response to MycER activation, while retaining a relatively normal glycolytic response (Fig 1*A* and 1*B*), appears to be the result of a more generalized mitochondrial dysfunction that includes an inability to accrete mass, to generate ROS, to maintain normal levels of TCA cycle intermediates and to properly regulate ETC function (Figs 1*C*, 2, 3 and S3 fig). Perhaps not surprisingly, these abnormalities were collectively associated with lower levels of ATP and higher levels of AMP, both in the basal state and in response to MycER activation (Fig 1*D and* 6*A*, & S1*B Fig*). The increased basal glycolytic rate of KO cells (Fig 1*A*), may therefore only reflect a partially effective compensatory mechanism aimed at correcting their relative energy deficit (Fig 1*D* and S1*B* Fig). Because Myc’s promotion of the pro-anabolic and proliferative states is tightly coupled to increased mitochondrial mass and function [20], it seems possible that the previously reported transformation-resistance of AMPK-deficient cells is a consequence of their ATP deficit and/or their mitochondrial unresponsiveness, despite their ability to up-regulate aerobic glycolysis (Fig 1*A*) [36, 75, 86, 87]. This idea has particular appeal given that numerous proliferative signaling pathways converge upon Myc whose uninterrupted expression is critical for maintaining tumor cell proliferation *in vivo* [88–90]. Thus, the seemingly paradoxical finding that AMPK suppression enhances proliferation yet confers transformation-resistance [36, 86, 87] can perhaps best be explained by postulating that the relative importance of AMPK on metabolism and proliferation may vary depending on Myc’s level of expression and/or deregulation. Over-expression of Myc might therefore cooperate with AMPK’s tendency to enhance glycolysis and Oxphos while simultaneously overriding its suppression of proliferation and the Warburg effect. This could be particularly useful under the most highly proliferative conditions where high anabolic demands could compromise and perhaps outstrip ATP stores (Fig 1*E*). AMPK activation might then cooperate with Myc to enhance both glycolysis and Oxphos without exerting deleterious effects on proliferation and its reliance on the Warburg effect. The relative resistance of AMPK-depleted cells to transformation could therefore reflect the cooperative nature of AMPK and Myc on these metabolic pathways.

Our transcriptional, proteomic and functional profiling underscores the significant differences that distinguish WT and KO cells both in the basal state and in response to Myc de-regulation (Figs 3 and 4). Indeed, we note that more genes serving as regulators of mitochondrial-specific functions such as DNA replication, maintenance of genomic integrity and transcription were found to be AMPK-responsive than Myc-responsive (13 of 20 vs. 8 of 20) (Fig 3*C*). It seems plausible that the widespread abnormalities in mitochondrial protein abundance and function described here are the ultimate consequence of this aberrant transcriptional regulation. Both the AMPK α1 and α2 subunits have been noted to localize to the nucleus as well as to the cytoplasm and a direct role for AMPK in transcriptional regulation has been suggested [91–93]. Moreover, a number of transcription factors, including PGC-1α and the carbohydrate response element binding protein, chREBP, which cooperates with Myc to regulate certain glucose-responsive genes [94] have been identified as putative AMPK targets [95–98]. Thus it is feasible that AMPK also alters many of the genes described here indirectly by virtue of its effects on key transcription factors many if not all of which are also direct Myc targets. Indeed, we note that several of the mitochondrial-related transcripts studied in Fig 3*C* such as PPARγ, POLRMT, SIRT3, and UCP2 are completely discordant with respect to their Myc responsiveness in WT and KO cells. The unresponsiveness of KO cell mitochondria appears to reflect a generalized loss of normal coordination among these various factors that is apparent both in their basal state and in response to Myc de-regulation. Obvious targets for AMPK-mediated post-translational modification include any of the large number of members that comprise the chromatin-modifying and transcriptional-enhancing protein complex that assembles in response to DNA binding by Myc [99, 100].

### Co-operativity between Myc and AMPK in determining cellular redox state

Maintaining a reduced intracellular environment is believed to protect against excessive oxidation of free thiols and other groups, particularly those residing within the catalytic domains of critical enzymes [44]. Our finding that both cytoplasmic and mitochondrial compartments, particularly the former, were more oxidized in KO cells under basal conditions supports the idea that redox regulation is an important, although likely indirect, AMPK function. The additional observation that cytoplasmic oxidation increased equivalently in WT and KO cells in response to MycER activation suggests that AMPK is less important in this compartment for regulating this balance following sudden oxidative stress. That WT mitochondria became more reduced following MycER activation further indicates that the cytoplasmic and mitochondrial compartments are under distinct forms of redox regulation. Multiple, non-mutually exclusive mechanisms could explain these findings including differences in the levels of various factors that maintain the reduced state such as thioredoxins, manganese superoxide dismutase, peroxiredoxins and the NAD+/NADH ratio [101–103]. The failure of mitochondria to significantly alter their redox state following MycER activation likely reflects their general unresponsiveness as discussed above.

The inter-membrane space is one source of ROS, which arise primarily as a consequence of electron leakage across Complexes I and III of the ETC [104, 105]. Myc-mediated enhancement of Oxphos may increase the absolute amount of this leakage without compromising ETC efficiency or integrity. The mitochondrial matrix is a second source of ROS, which originate from qualitative defects in ETC function as occurs in patients with respiratory chain mutations [105–107]. The compartmentalization of ROS from these two sources might well have different effects on the redox state, with the former source tending to contribute more to cytoplasmic oxidation and the latter to mitochondrial. MycER activation likely favors the generation of ROS via the first mechanism, and such ROS might be less able to oxidize roGFP residing in the matrix.

### Changes in PK and PDH as a potential mechanism for metabolite differences between WT and KO MEFs

In response to MycER activation, WT cells underwent a redistribution of metabolic substrates that included the accumulation of selective glycolytic intermediates and a depletion of TCA cycle intermediates (Fig 6*A*). One explanation for these findings is an increased reliance on the Warburg effect, which is often accompanied by a shift from the M1 to the M2 PK isoform. The latter possesses a lower affinity for PEP thus slowing its conversion to pyruvate and allowing more time for the accumulation of upstream substrates and their diversion into anabolic pathways [16, 77, 79, 82, 83]. Although we did not detect significant changes in the PKM1:PKM2 ratio upon MycER activation in WT cells (S7 Fig), we did observe a decrease in total PK activity (Fig 6*D*). This by itself could explain the accumulation of substrates upstream of PEP thereby limiting the conversion of pyruvate to acetyl CoA. It could also account for the observed depletion of TCA cycle intermediates in response to the Myc-mediated increase in Oxphos (Fig 6*A*). A second possibility is that the enhanced glycolysis mediated by MycER activation in WT cells supplies sufficient levels of substrates for both the Warburg effect and Oxphos. This could explain the increased production of lactate and accompanying extracellular acidification in response to MycER (Fig 1*A*). Taken together, increased glycolysis, an overall buildup of intermediates due to reduced PK, combined with increased mitochondrial activity and a depletion of TCA cycle substrates, could explain many, if not all, of the metabolite shifts detected in WT cells in response to Myc activation. However, this change is not without its energetic costs as evidenced by the accompanying chronic ATP depletion, higher levels of AMP and AMPK-activation (Figs 1*D*, 1*E* and 6*A*).

KO cells in their basal state were relatively depleted of glycolytic substrates and oversupplied with TCA cycle intermediates compared to WT cells (Fig 6*A*). Although the activities of PK and PDH were substantially different between these 2 cell lines (Fig 6*C* and 6*D*), the fact that levels of PEP, pyruvate and AcCoA were actually quite similar (Fig 6*A* and 6*E*) argues against the idea that significant changes in any of these could account for the differential levels and distribution of downstream TCA cycle substrates. Rather, the accumulation of TCA cycle intermediates most likely reflects the consequences of the loss of AMPK in these cells as manifested by their inability to properly coordinate the mitochondrial response. The ultimate result is even lower levels of ATP and higher levels of AMP in KO cells both in the resting state and in response to MycER activation (Figs 1*D* *and* 6*A* & S1*B Fig*).

Cross-talk between Myc and AMPK. The wide-ranging differences between WT and KO cells in response to MycER activation reported here demonstrate that Myc and AMPK likely engage in a complex cross-talk, the presumed purpose of which is to correctly balance anabolic and proliferative needs with cellular energy levels (Fig 7). The need for maintaining this balance is most dramatically illustrated by the rapid and dramatic reduction of ATP levels that follow MycER activation in WT cells and by the even steeper decline in KO cells (Fig 1*D* and S1B Fig). This ATP-mediated cross-talk between AMPK and Myc has been previously demonstrated in *myc-/-* rat fibroblasts in which AMPK is constitutively activated as a result of the inability to maintain normal levels of ATP due to the overall failure of Myc-dependent glycolysis and Oxphos [20, 23]. Thus, balancing ATP production and AMPK activation and with anabolic demands is a process that occurs in response to both Myc over- and under-expression.

Other potential mediators of the cross-talk between Myc and AMPK, that are largely independent of but linked to ATP levels, are ROS which, in the examples provided here, contribute extensively to AMPK activation (Fig 1*G*). ROS are well-known second messengers that are rapidly generated in response to many different growth stimuli and the large increase in ETC function that accompanies Myc over-expression [20, 48, 108, 109]. Their ability to maintain AMPK in an active state, even beyond the point at which ATP levels have normalized (Fig 1*E*-1*H*) suggests that ROS might serve to limit growth factor-mediated proliferation at a point before which ATP levels are compromised and might also serve to more rapidly restore ATP levels to normal following a proliferative signal.

In retrospect, a close relationship between Myc and AMPK might have been anticipated given their well-known regulation of a variety of similar processes as noted above. A similar relationship has also been previously suggested by Liu *et al*. [110] who showed that ARK5, an upstream regulator of AMPK, could also affect several cellular functions that are relevant to Myc. These included the ability of ARK5 to protect against Myc-mediated apoptosis, to increase oxygen consumption, to negatively regulate cell size and to promote cell cycle progression. However, many of the consequence of ARK5 suppression in cancer cells were not observed in the current study. Perhaps most importantly, and unlike the case with AMPK described here, the depletion of ARK5 in transformed cells had little influence on the expression of Myc target genes [110]. Taken together, these observations suggest that, while ARK5 and AMPK operate within the same pathway, their communication with Myc may occur by distinct mechanisms, may be cell-type specific and may serve distinct purposes.

## Supporting Information

S1 Fig *(A)* Immunoblots of endogenous c-Myc and MycER in AMPK WT and KO MEFs before and after MycER transduction and β-actin loading control. Both proteins were detected with an anti-Myc antibody. *(B)* Baseline ATP levels in WT and KO cells. The results represent the data obtained in Fig 1*D* plotted as absolute rather than relative ATP levels. Several repeat experiments showed ATP levels in KO cells to be reproducibly lower than WT cells by 30–40%.
(TIF)

S2 Fig Seahorse Flux Analysis of Extracellular Acidification Rate (ECAR) and Oxygen Consumption Rate (OCR). Cells were plated and analyzed as described in Materials and Methods. (A) ECAR normalized to cell number at conclusion of the experiment, which includes the addition of 1 μM oligomycin (oligo, an inhibitor of Complex V [ATP synthase]), 0.3 μM carbonyl cyanide-p-trifluoromethoxyphenylhydrazone (FCCP, an uncoupling agent), 100 mM 2-Deoxy-D-glucose (2-DG, an inhibitor of glycolysis), and 1 μM rotenone (rot, a complex I inhibitor). Note that the addition of oligomycin is associated with a continued higher level of ECAR by both WT+Myc and KO+Myc cells, which is consistent with their overall rates of glycolysis being enhanced following MycER activation (B) Representation of basal respiration, glycolytic capacity, non-glycolytic acidification, and glycolytic reserve (differences in respiration after addition of oligomycin). (C) OCR normalized to cellular number at the conclusion of the experiment, including the same set of injections described in (A). (D) Representation of OCR basal respiration, ATP-dependent respiration, maximum respiration, non-mitochondrial respiration, and the spare respiratory capacity.
(TIF)

S3 Fig Quantification of the results shown in Fig 2. *In situ* assays for each complex were performed on triplicate samples. Results are expressed as the mean ± 1 SEM after normalizing each sample’s activity to the amount of protein present in the respective complex. This value in WT cells was arbitrarily set to 1 in all cases to allow for relative comparisons. Complex II could not be reliably assayed *in situ* and was therefore assayed in separate reactions and adjusted to total input mitochondrial protein content (see Fig 2*C*). Significance was determined using the Students’ t-test and all values are compared to WT cells.
(TIF)

S4 Fig Quantification of real time qRT-PCR data depicted in Fig 3*A*. The values presented were compared to average gene expression and normalized to β_2_ microglobulin gene expression.**** *P* < 0.0001, *** *P* < 0.001, ** *P* < 0.01, * *P* < 0.05.
(TIF)

S5 Fig Quantification of real time qRT-PCR data depicted in Fig 3*D*. The values presented were compared to average gene expression and normalized to β_2_ microglobulin gene expression.**** *P* < 0.0001, *** *P* < 0.001, ** *P* < 0.01, * *P* < 0.05.
(TIF)

S6 Fig Isotope distribution. High-resolution dMS chromatogram (top) and mass spectrum (bottom) showing the isotope distribution for the tryptic peptide ATEMVEVGPEDDEVGAERGEATDLLR derived from Polymerase I and transcript release factor (Ptrf) with monoisotopic m/z = 930.103 Da and retention time 43.5 minutes. Colored lines show the average signal for 4 WT (blue), 4 KO (red), 4 WT+Myc (green), 4 KO+Myc (pink), and 6 pooled control (tan) samples.
(TIF)

S7 Fig Immuno-blotting for selected pyruvate metabolizing enzymes. Pyruvate dehydrogenase (PHDE) and Ser_293_ (activated) phosphorylated PDHE. Pyruvate dehydrogenase kinase (PDK1), Pyruvate dehydrogenase phosphatase (PDP2), Pyruvate kinase M1 and M2 (PKM1/2), and β-actin loading control.
(TIF)

S1 Table qRT-PCR primers used in the current study. (DOCX)

S2 Table Antibodies used in the current study. (DOCX)

S3 Table 345 mitochondrial proteins identified by LC-MS/MS analysis. Protein name, including the organism name (OS), gene name (GN), protein existence (PE, a numerical value describing the evidence of existence for the protein) and sequence version (SV). Gene name is how the protein is identified throughout the paper, followed by the primary accession number for reference. Overall p-value is calculated by a two way ANOVA. p- and q-values<0.05 are highlighted in red text throughout the table. The mean protein intensities are prepared and run in 4 individual samples for each cell type. Fold change, p-value and false discovery rate (q-value) were calculated as described in Statistical Analysis and the selected features are identified by blue text in the fold change columns. Features were selected by a conservative cut off of q<0.05, with the exception of the comparison of AMPK WT to KO. KO proteins had an overall slightly higher average intensity, so to reduce potential bias, proteins with greater abundance in KO but with fold change less than 2.6 (twice the fold change of overall mitochondrial abundance in KO samples) were not considered.
(XLSX)
