# Supplementary figures and images for: c-Myc and AMPK Control Cellular Energy Levels by Cooperatively Regulating Mitochondrial Structure and Function
Source: PLoS One. 2015 Jul 31;10(7):e0134049. doi: 10.1371/journal.pone.0134049 (PMC4521957; doi:10.1371/journal.pone.0134049)

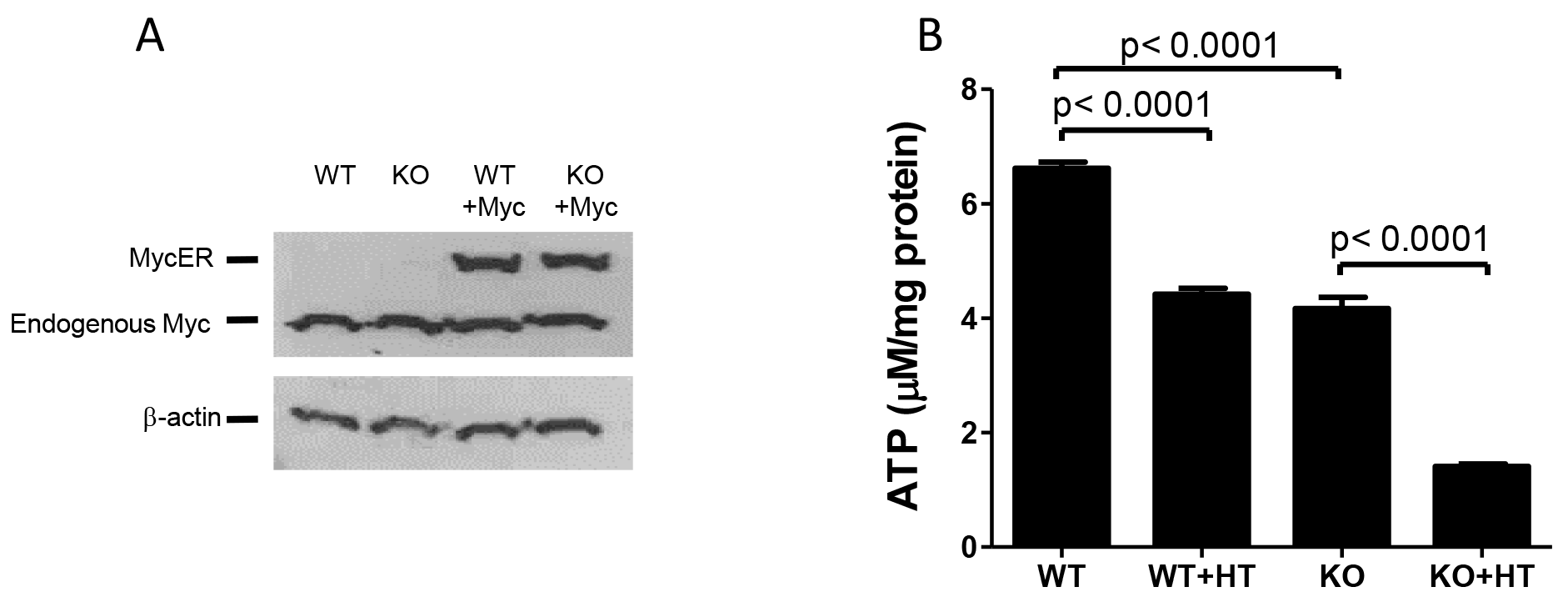

Supplement: S1 Fig — (A) Immunoblots of endogenous c-Myc and MycER in AMPK WT and KO MEFs before and after MycER transduction and β-actin loading control. Both proteins were detected with an anti-Myc antibody. (B) Baseline ATP levels in WT and KO cells. The results represent the data obtained in Fig 1D plotted as absolute rather than relative ATP levels. Several repeat experiments showed ATP levels in KO cells to be reproducibly lower than WT cells by 30–40%. (TIF) [file pone.0134049.s001.tif]

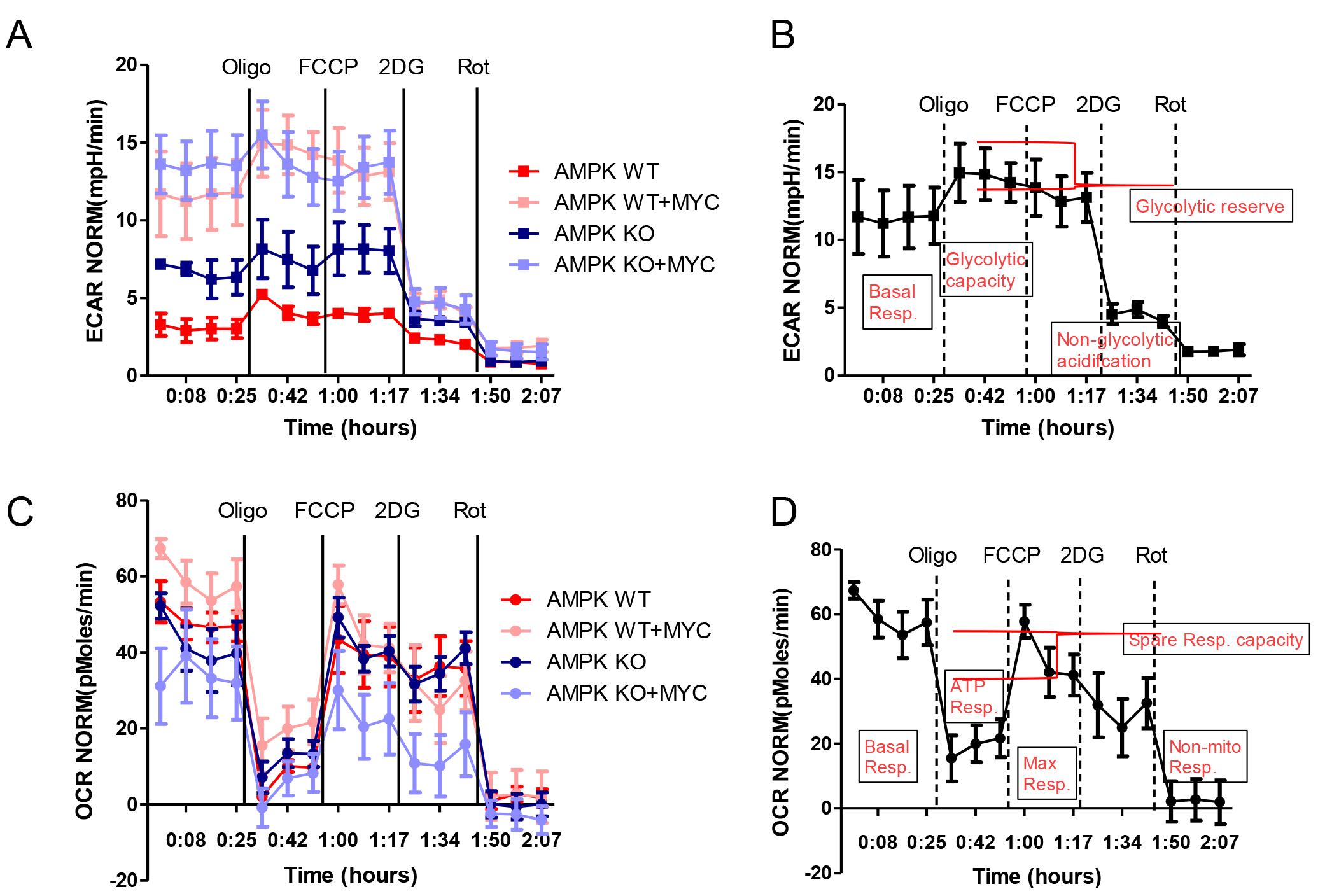

Supplement: S2 Fig — Cells were plated and analyzed as described in Materials and Methods. (A) ECAR normalized to cell number at conclusion of the experiment, which includes the addition of 1 μM oligomycin (oligo, an inhibitor of Complex V [ATP synthase]), 0.3 μM carbonyl cyanide-p-trifluoromethoxyphenylhydrazone (FCCP, an uncoupling agent), 100 mM 2-Deoxy-D-glucose (2-DG, an inhibitor of glycolysis), and 1 μM rotenone (rot, a complex I inhibitor). Note that the addition of oligomycin is associated with a continued higher level of ECAR by both WT+Myc and KO+Myc cells, which is consistent with their overall rates of glycolysis being enhanced following MycER activation (B) Representation of basal respiration, glycolytic capacity, non-glycolytic acidification, and glycolytic reserve (differences in respiration after addition of oligomycin). (C) OCR normalized to cellular number at the conclusion of the experiment, including the same set of injections described in (A). (D) Representation of OCR basal respiration, ATP-dependent respiration, maximum respiration, non-mitochondrial respiration, and the spare respiratory capacity. (TIF) [file pone.0134049.s002.tif]

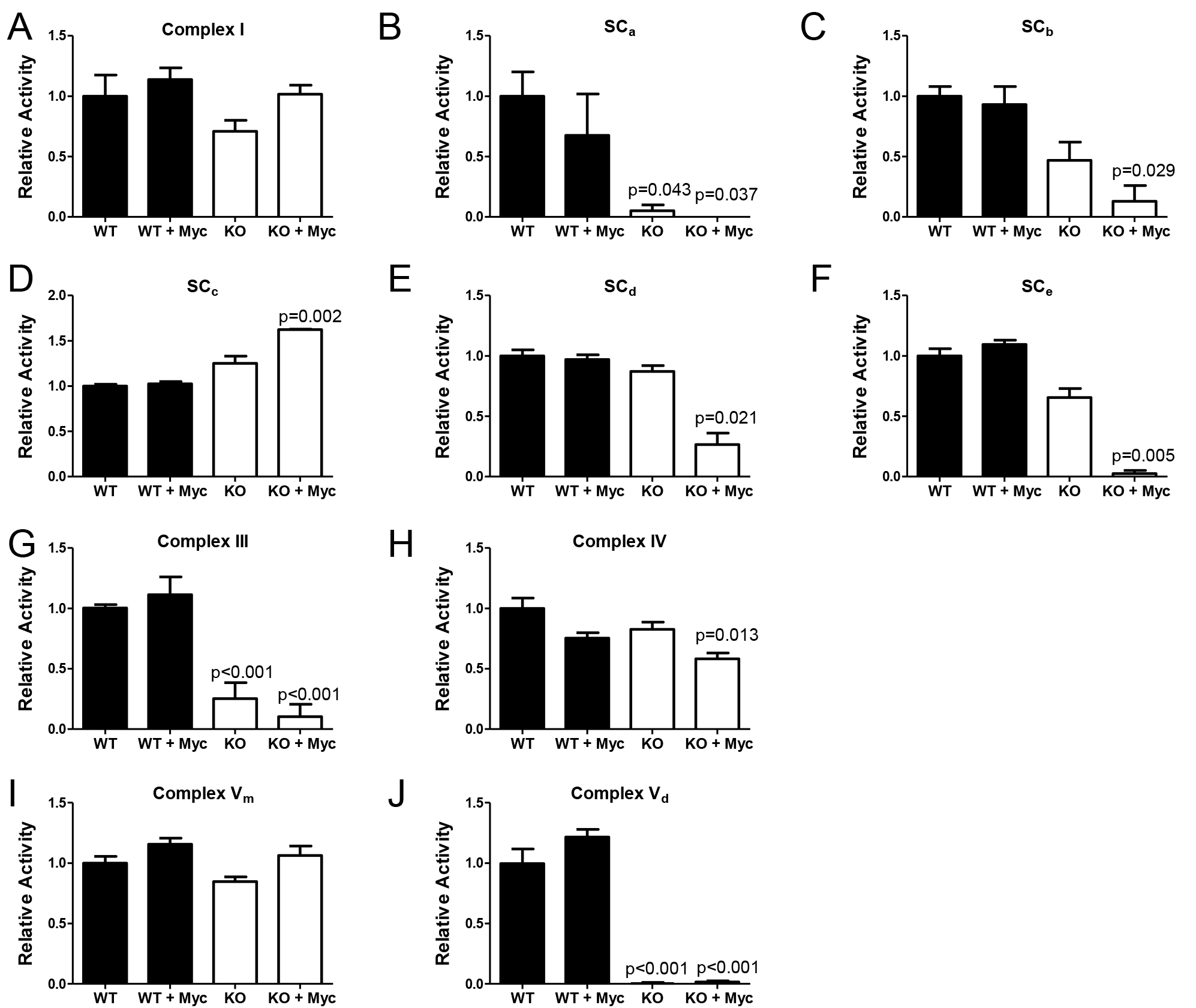

Supplement: S3 Fig — In situ assays for each complex were performed on triplicate samples. Results are expressed as the mean ± 1 SEM after normalizing each sample’s activity to the amount of protein present in the respective complex. This value in WT cells was arbitrarily set to 1 in all cases to allow for relative comparisons. Complex II could not be reliably assayed in situ and was therefore assayed in separate reactions and adjusted to total input mitochondrial protein content (see Fig 2C ). Significance was determined using the Students’ t-test and all values are compared to WT cells. (TIF) [file pone.0134049.s003.tif]

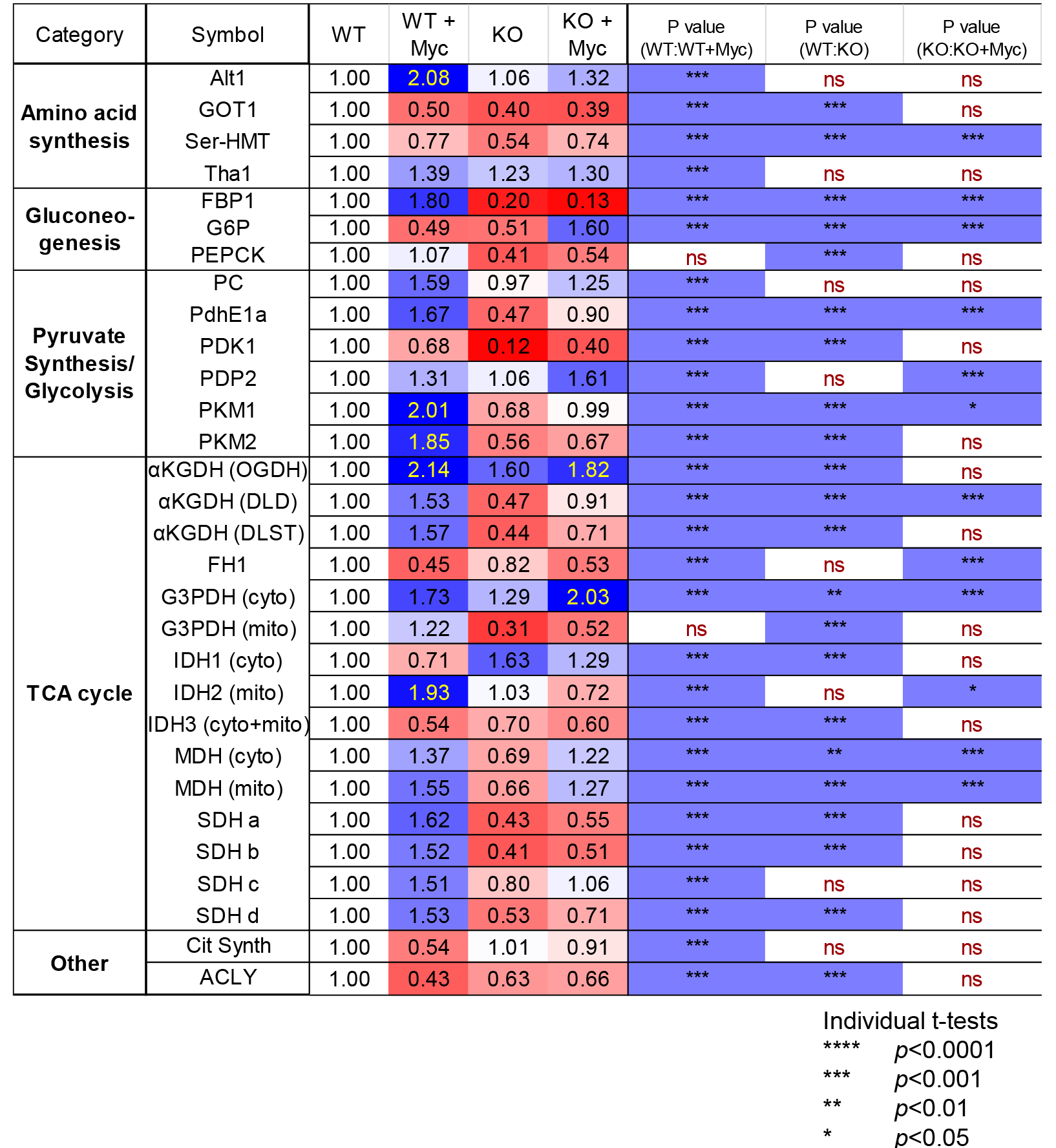

Supplement: S4 Fig — The values presented were compared to average gene expression and normalized to β2 microglobulin gene expression.**** P < 0.0001, *** P < 0.001, ** P < 0.01, * P < 0.05. (TIF) [file pone.0134049.s004.tif]

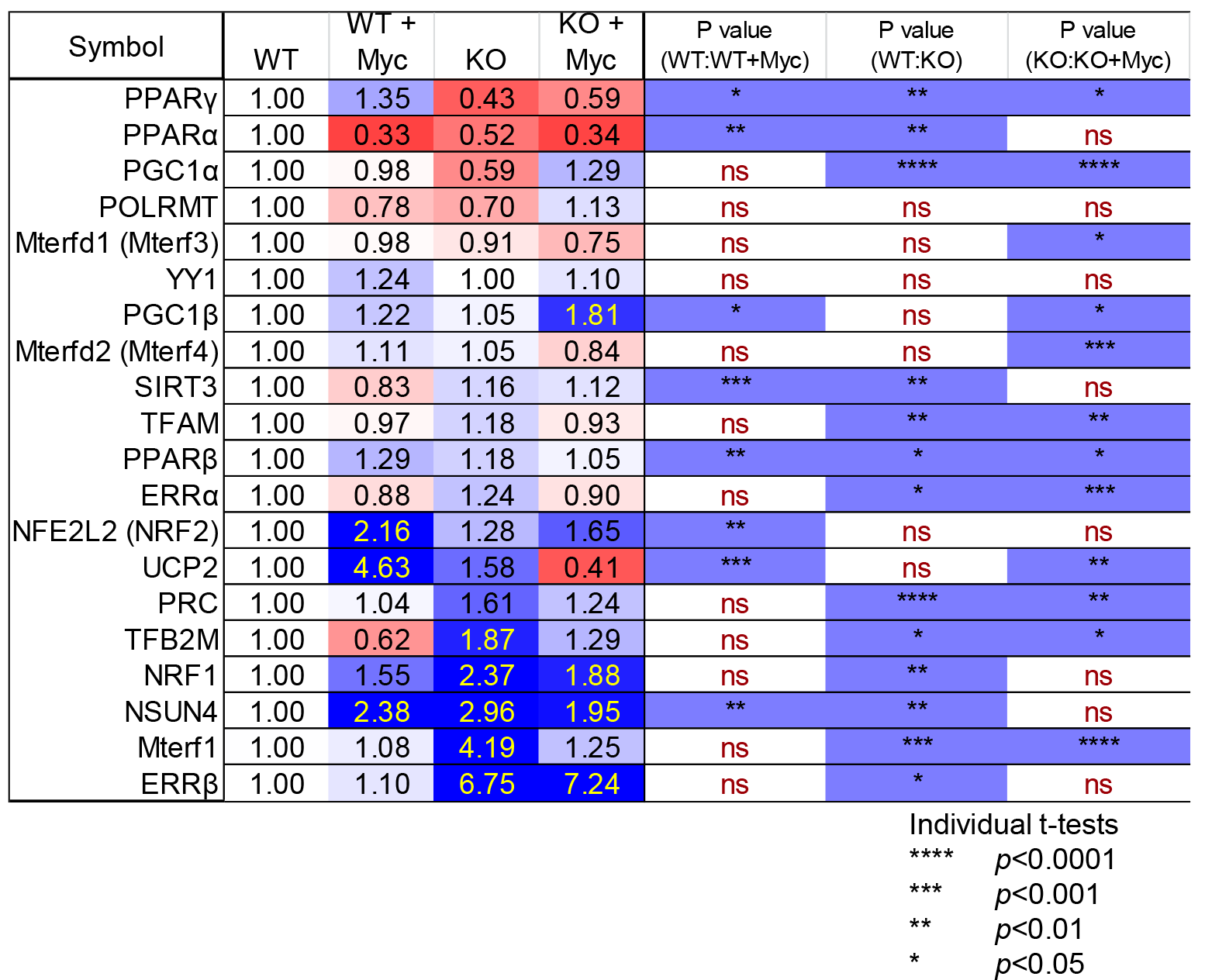

Supplement: S5 Fig — The values presented were compared to average gene expression and normalized to β2 microglobulin gene expression.**** P < 0.0001, *** P < 0.001, ** P < 0.01, * P < 0.05. (TIF) [file pone.0134049.s005.tif]

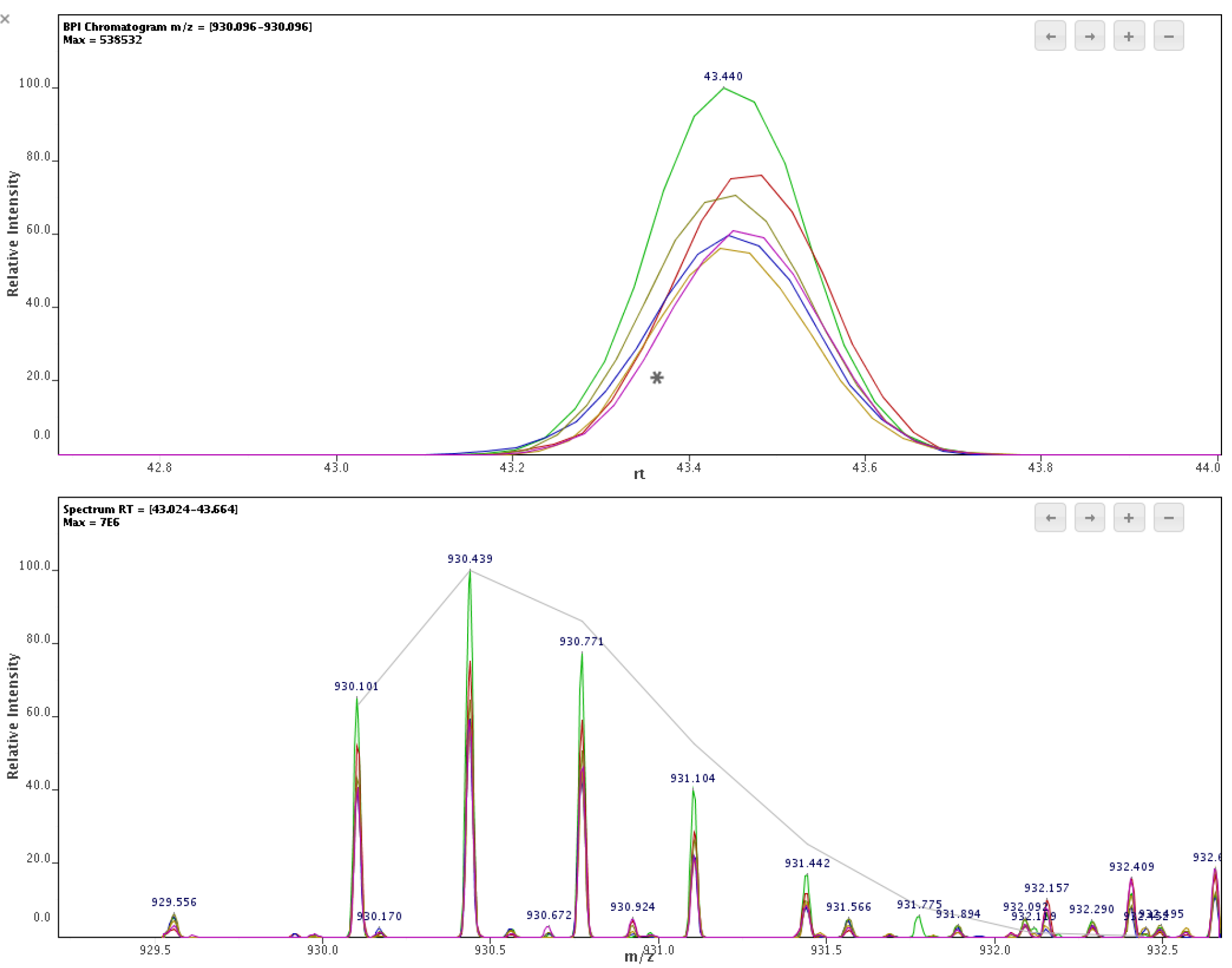

Supplement: S6 Fig — High-resolution dMS chromatogram (top) and mass spectrum (bottom) showing the isotope distribution for the tryptic peptide ATEMVEVGPEDDEVGAERGEATDLLR derived from Polymerase I and transcript release factor (Ptrf) with monoisotopic m/z = 930.103 Da and retention time 43.5 minutes. Colored lines show the average signal for 4 WT (blue), 4 KO (red), 4 WT+Myc (green), 4 KO+Myc (pink), and 6 pooled control (tan) samples. (TIF) [file pone.0134049.s006.tif]

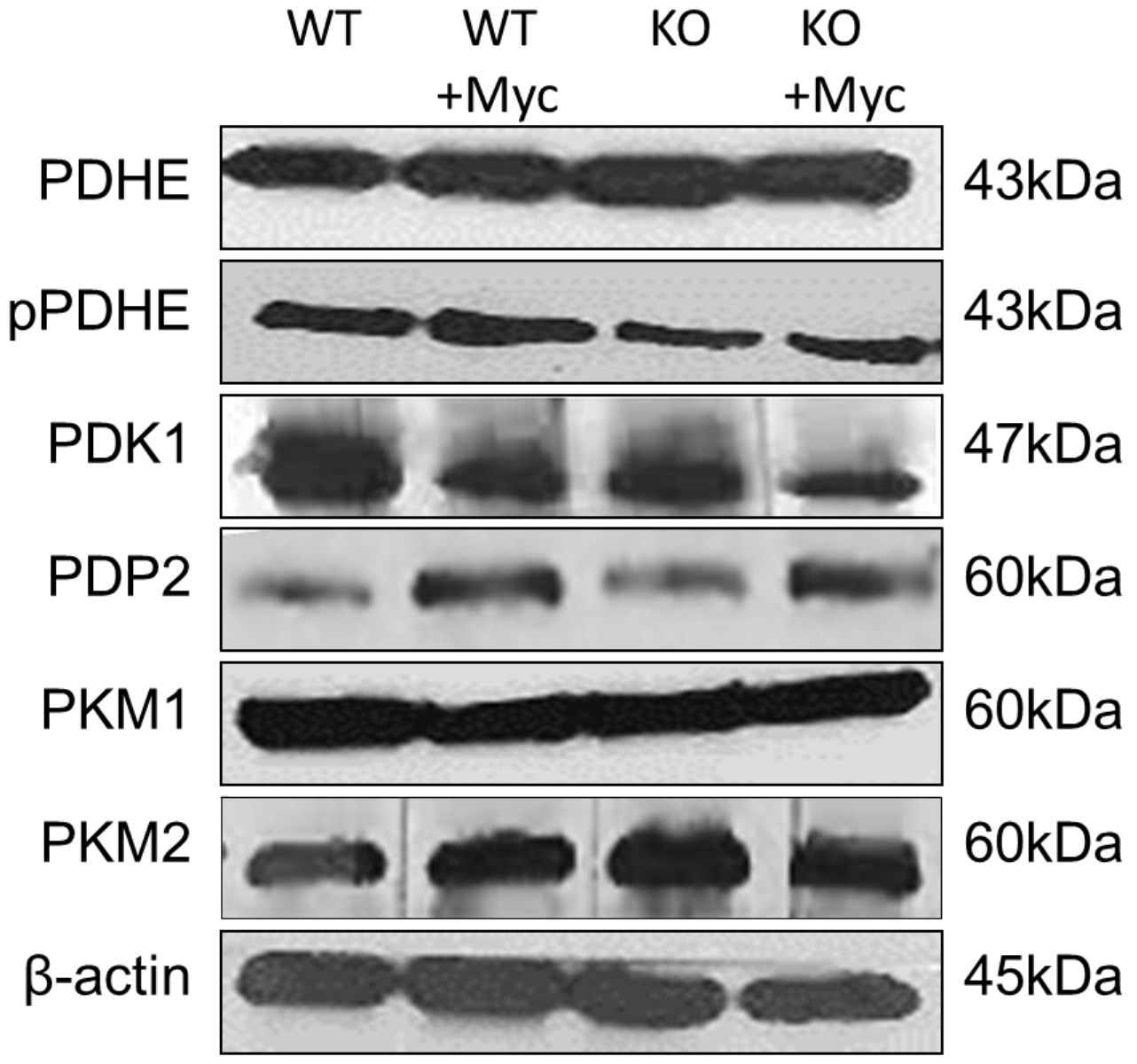

Supplement: S7 Fig — Pyruvate dehydrogenase (PHDE) and Ser293 (activated) phosphorylated PDHE. Pyruvate dehydrogenase kinase (PDK1), Pyruvate dehydrogenase phosphatase (PDP2), Pyruvate kinase M1 and M2 (PKM1/2), and β-actin loading control. (TIF) [file pone.0134049.s007.tif]
