# Supplementary material for: c-Myc and AMPK Control Cellular Energy Levels by Cooperatively Regulating Mitochondrial Structure and Function
Source: PLoS One. 2015 Jul 31;10(7):e0134049. doi: 10.1371/journal.pone.0134049 (PMC4521957; doi:10.1371/journal.pone.0134049)
Supplement: S1 Table — (DOCX) [file pone.0134049.s008.docx]

Table S1. qRT-PCR Primers

| **Symbol:** | **Description (mRNA):** | **Accession** | **Forward Primer Sequence** | **Reverse Primer Sequence** | **Annealing** | **Product** |
| --- | --- | --- | --- | --- | --- | --- |
|  |  | **ID:** | **(5' → 3')** | **(5' → 3')** | **Temp.** | **Length** |
|  |  |  |  |  |  | **(bps)** |
| ACLY | ATP citrate lyase (Acly) | BC056378.1 | ACCCTTTCACTGGGGATCACA | GACAGGGATCAGGATTTCCTTG | 56 | 65 |
| Alt1 | Alanine Aminotransferase1 | BK005127.1 | TCCAGGCTTCAAGGAATGGAC | CAAGGCACGTTGCACGATG | 55 | 113 |
| Cit Synth | citrate synthase | BC029754.1 | GGACAATTTTCCAACCAATCTGC | TCGGTTCATTCCCTCTGCATA | 55 | 109 |
| ERRα | estrogen related receptor, alpha | NM_007953 | GCCTCCAATGAGTGTGAGATC | TTTGTACTTCTGCCGTCCG | 60 | 138 |
| ERRβ | estrogen related receptor, beta | NM_011934 | CATGAAATGCCTCAAAGTGGG | AAATCGGCAGGTTCAGGTAG | 60 | 125 |
| FBP1 | fructose 1,6-bisphosphatase 1 | NM_019395 | GACTGGGGATCAAGTAAAGAAGC | AGGTAGCGTAGGACGACTTCA | 55 | 80 |
| FH1 | Fumerate Hydratase | NM_010209.2 | GAATGGCAAGCCAAAATTCCTT | TCTTACGGTCTGAGCACCATAA | 55 | 95 |
| G3PDH (cyto) | cytosolic glycerol-3-phoshate dehydrogenase 1 (GPD1) | NM_010271 | ATGGCTGGCAAGAAAGTCTG | CCTGCATTGCTACCCACGAT | 51 | 80 |
| G3PDH (mito) | mitochondrial glycerol-3-phoshate dehydrogenase 1 (GPD2) | U60987 | ATGGCGTTTCAAAAGGCAGTG | ACGGAGGAGGTCCCAAAACAG | 51 | 79 |
| G6P | glucose-6-phosphatase (catalytic) | U00445.1 | CGACTCGCTATCTCCAAGTGA | GGGCGTTGTCCAAACAGAAT | 60 | 208 |
| GOT1 | asprate aminotransferase isoenzyme, glutamine oxaloacetate transaminase | J02623.1 | GCGCCTCCATCAGTCTTTG | ATTCATCTGTGCGGTACGCTC | 55 | 133 |
| IDH1 (cyto) | isocitrate dehydrogenase - cytosolic | NM_001111320.1 | ATGCAAGGAGATGAAATGACACG | GCATCACGATTCTCTATGCCTAA | 55 | 116 |
| IDH2 (mito) | isocitrate dehydrogenase 1 (NADP+) mitochondrial (IDH2) | U51167 | AAGAGCCCTAACGGAACGAT | TCTTTGGGGTGAAGACCAAC | 51 | 202 |
| IDH3 (cyto+mito) | isocitrate dehydrogenase3 - mito, (NAD+), alpha | NM_029573 | TGGGTGTCCAAGGTCTCTC | CTCCCACTGAATAGGTGCTTTG | 55 | 177 |
| MDH (cyto) | cytosolic malate dehydrogenase (MDH1) | NM_008618 | GAACCAATCAGAGTCCTTGTGAC | GGCACAGTCTTGCAGTTCCA | 51 | 177 |
| MDH (mito) | mitochondrial malate dehydrogenase (MDH2) | NM_008617 | GCAACCCCTTTCACTCCTG | TCTGGTCTCAATGTGACTCAGAT | 51 | 112 |
| Mterf1 | Mitochondrial transcription termination factor 1a | NM_001013023.2 | GTTCCTTTGCTCTGTTGGATTG | GAAAGCAGCCTCTCTCTTATGT | 60 | 363 |
| Mterfd1 (Mterf3) | MTERF domain containing 1 | NM_025547.3 | TCATCGTCAAGTTTCCACAGT | AGGTTTTGCTGGGTCATACTG | 60 | 107 |
| Mterfd2 (Mterf4) | MTERF domain containing 2 | NM_178051.3 | CAGATGCCCCACTGTTTTG | TGTGTCTCTGCTTGATCTTGG | 60 | 146 |
| NFE2L2 (NRF2) | Nuclear factor, erythroid derived 2, like 2 | NM_010902.3 | TCCCATTTGTAGATGACCATGAG | CCATGTCCTGCTCTATGCTG | 60 | 150 |
| NRF1 | nuclear respiratory factor 1 (Nrf1) | NM_010938 | AATGTCCGCAGTGATGTCC | GCCTGAGTTTGTGTTTGCTG | 60 | 149 |
| NSUN4 | NOL1/NOP2/Sun domain family, member 4 | NM_028142.4 | CCAAGTCCGAGTTACCTCATG | TCTTCCTTGACCGCTGAAAG | 60 | 149 |
| PC | Pyruvate carboxylase | L09192.1 | CTGAAGTTCCAAACAGTTCGAGG | CGCACGAAACACTCGGATG | 55 | 162 |
| PdhE1a | pyruvate dehydrogenase E1 alpha 1 (Pdha1), mitochondrial protein | NM_008810.2 | GAAATGTGACCTTCATCGGCT | TGATCCGCCTTTAGCTCCATC | 55 | 123 |
| PDK1 | Pyruvate dehydrogenase kinase, isoenzyme 1, mitochondrial protein | NM_172665 | GGACTTCGGGTCAGTGAATGC | TCCTGAGAAGATTGTCGGGGA | 56 | 122 |
| PDP2 | pyruvate dehydrogenase phosphatase regulatory subunit (Pdpr) | NM_198308.1 | AAGACAAAGGACTAGCCCAGG | GATAGGCCACGGATGTACCC | 55 | 146 |
| PEPCK | phosphoenolpyruvate carboxykinase 2, mitochondrial (PCK2), mitochondrial protein | NM_028994.2 | ATGGCTGCTATGTACCTCCC | GCGCCACAAAGTCTCGAAC | 55 | 148 |
| PGC1α | peroxisome proliferative activated receptor, gamma, coactivator 1 alpha (Ppargc1a) | NM_008904 | CACCAAACCCACAGAAAACAG | GGGTCAGAGGAAGAGATAAAGTTG | 60 | 125 |
| PGC1β | peroxisome proliferative activated receptor, gamma, coactivator 1 beta (Ppargc1b) | NM_133249 | GGTGTTCGGTGAGATTGTAGAG | GTGATAAAACCGTGCTTCTGG | 60 | 72 |
| PKM1 | Pyruvate Kinase, Muscle, isoform 1 | NM_01253883 | TTGTGCGAGCCTCCAGTC | ACTCCGTGAGAACTATCAAAGC | 55 | 106 |
| PKM2 | Pyruvate Kinase, Muscle, isoform 2 | NM_011099 | TTGCAGCTATTCGAGGAACTCCG | CACGATAATGGCCCCACTGC | 55 | 115 |
| POLRMT | Polymerase (RNA) mitochondrial (DNA directed) | NM_172551 | AGAAGCCCAACACTCTGAAG | ATGTGTCCAGAAGCAGTCG | 60 | 146 |
| PPARα | peroxisome proliferator activated receptor alpha | NM_011144 | CATTTCCCTGTTTGTGGCTG | ATCTGGATGGTTGCTCTGC | 60 | 133 |
| PPARβ | peroxisome proliferator activator receptor delta | NM_011145 | GGAAAAGTTTTGGCAGGAGC | TGTCTTCATCTGTCAGTGAGC | 61 | 149 |
| PPARγ | peroxisome proliferator activated receptor gamma | NM_011146 | ATAGGTGTGATCTTAACTGCCG | CCAACAGCTTCTCCTTCTCG | 60 | 147 |
| PRC | peroxisome proliferative activated receptor, gamma, coactivator-related 1 | NM_001081214 | ACTCAGGCATTGACATTCCC | TTTCGCCAAGAGTGAGACAG | 61 | 146 |
| SDH a | Succinate dehydrogenase subunit A | NM_023281 | GGAACACTCCAAAAACAGACCT | TCCACCACTGGGTATTGAGTAG | 51 | 106 |
| SDH b | Succinate dehydrogenase subunit B | NM_023374 | ATTTACCGATGGGACCCAGAC | GTCCGCACTTATTCAGATCCAC | 51 | 79 |
| SDH c | Succinate dehydrogenase subunit C | NM_025321 | GCTGCGTTCTTGCTGAGACACA | ATCTCCTCCTTAGCTGTGGTT | 51 | 110 |
| SDH d | Succinate dehydrogenase subunit D | NM_025848 | TGGTCAGACCCGCTTATGTG | GAGCAGGGATTCAAGTACCCA | 51 | 193 |
| Ser-HMT | Serine Hydromethyltransferase | AF237702.1 | CAGGGCTCTGTCTGATGCAC | CGTAACGCGCTCTTGTCAC | 57 | 91 |
| SIRT3 | NAD-dependent deacetylase sirtuin-3, mitochondrial | NM_022433 | CGGCTCTATACACAGAACATCG | CATCAGCCCATATGTCTTCCC | 61 | 145 |
| TFAM | Transcription factor A, mitochondrial | NM_009360 | CACCCAGATGCAAAACTTTCAG | CTGCTCTTTATACTTGCTCACAG | 58 | 147 |
| TFB2M | Transcription factor B2, mitochondrial | NM_008249 | ACCAAAACCCATCCCGTC | TCTGTAAGGGCTCCAAATGTG | 60 | 141 |
| Tha1 | Threonine aldolase | NM_027919.4 | CTCAGTGGTCTAGGAATTGGGC | GTCTTCGCCGTAATCATCGTC | 55 | 137 |
| UCP2 | Mitochondrial Uncoupling Protein 2 | NM_011671 | GCATTGGCCTCTACGACTC | AAGCGGACCTTTACCACATC | 60 | 145 |
| YY1 | YY1 transcription factor | NM_009537 | GATACCTGGCATTGACCTCTC | ATAGCAGAGTTATCCCTGAACATC | 60 | 147 |
| αKGDH (DLD) | dihydrolipoamide dehydrogenase, mitochondrial protein | NM_007861.4 | GAGCTGGAGTCGTGTGTACC | CCTATCACTGTCACGTCAGCC | 55 | 138 |
| αKGDH (DLST) | dihydrolipoamide S-succinyltransferase (E2 component of 2-oxo-glutarate complex) | NM_030225.4 | GGAACTGCCCTCTAGGGAGA | GACGCTACCACTGTTAATGACC | 55 | 101 |
| αKGDH (OGDH) | alpha ketogluterate dehydrogenase (OGDH) | NM_001252282 | AGGGCATATCAGATACGAGGG | CTGTGGATGAGATAATGTCAGCG | 51 | 106 |
