# Supplementary material for: c-Myc and AMPK Control Cellular Energy Levels by Cooperatively Regulating Mitochondrial Structure and Function
Source: PLoS One. 2015 Jul 31;10(7):e0134049. doi: 10.1371/journal.pone.0134049 (PMC4521957; doi:10.1371/journal.pone.0134049)
Supplement: S2 Table — (DOCX) [file pone.0134049.s009.docx]

Table S2. Antibodies used in current study

| **Antibody ID** | **Name of antibody** | **Description** | **Host Species** |  | **Vendor** | **Catalog no.** | **dilution** |
| --- | --- | --- | --- | --- | --- | --- | --- |
| [AB_10694064](http://antibodyregistry.org/AB_10694064) | AMP-dependent protein kinase alpha (AMPK) | Polyclonal | Rabbit | IgG | Cell Signaling | 2532S | 1:1000 |
| [AB_2169396](http://antibodyregistry.org/AB_2169396) | pAMPK(Thr_172_) (D79.5E) | Monoclonal | Rabbit | IgG | Cell Signaling | 4188S | 1:1000 |
|  | Pyruvate dehydrogenase E1 subunit (PDH) (D6) | Monoclonal | Mouse | IgG2a | Santa Cruz | sc-377092 | 1:1000 |
| [AB_1084131](http://antibodyregistry.org/AB_1084131) | pPDH E1 (Ser_293_) | Polyclonal | Rabbit | IgG | EMD Millipore | AP1062 | 1:4000 |
| [AB_1904078](http://antibodyregistry.org/AB_1904078) | Pyruvate dehydrogenase kinase (PDK1) | Monoclonal | Rabbit | IgG | Cell Signaling | 3820S | 1:1000 |
| [AB_10662227](http://antibodyregistry.org/AB_10662227) | Pyruvate dehydrogenase phosphatase 2 (PDP2) | Polyclonal | Rabbit | IgG | Biovision | 3944-200 | 1:500 |
|  | Pyruvate kinase M1 isoform (PKM1) | Monoclonal | Rabbit | IgG | Cell Signaling | 7067S | 1:1000 |
| [AB_2252325](http://antibodyregistry.org/AB_2252325) | Pyruvate kinase M2 isoform (PKM2) | Polyclonal | Rabbit | IgG | Cell Signaling | 3198S | 1:1000 |
| [AB_2242334](http://antibodyregistry.org/AB_2242334) | β-actin | Monoclonal | Mouse | IgG2b | Cell Signaling | 3700S | 1:10000 |
